# Atonal homolog 8/Math6 regulates differentiation and maintenance of skeletal muscle

**DOI:** 10.3389/fcell.2022.950414

**Published:** 2022-08-16

**Authors:** Satya Srirama Karthik Divvela, Eric Bekoe Offei, Florian Suerland, David Revuelta García, Julia Kwiatkowski, Ajeesh Balakrishnan-Renuka, Pauline Bohne, Marion Böing, Gabriela Morosan-Puopolo, Melanie D. Mark, Beate Brand-Saberi

**Affiliations:** ^1^ Department of Anatomy and Molecular Embryology, Medical Faculty, Ruhr-University Bochum, Bochum, Germany; ^2^ University of Ghana, School of Veterinary Medicine, Legon, Ghana; ^3^ Department of Behavioral Neuroscience, Faculty of Biology and Biotechnology, Ruhr-University Bochum, Bochum, Germany

**Keywords:** hypoxia, atrophy, myostatin, AKT/mTOR, math6, regeneration, Atoh8, skeletal muscle

## Abstract

Atonal Homolog 8 (Atoh8) belongs to a large superfamily of transcriptional regulators called basic helix-loop-helix (bHLH) transcription factors. Atoh8 (murine homolog “Math6”) has been shown to be involved in organogenesis during murine embryonic development. We have previously identified the expression of Atoh8 during skeletal myogenesis in chicken where we described its involvement in hypaxial myotome formation suggesting a regulatory role of Atoh8 in skeletal muscle development. Within the current study, we analyzed the effect of the loss of function of Atoh8 in murine primary myoblasts and during differentiation of pluripotent stem cells into myotubes, and the effect of its gain of function in C2C12 cells. Based on the observed results, we conclude that Atoh8 regulates myoblast proliferation via modulating myostatin signaling. Further, our data revealed a reduced muscle mass, strength and fiber size with significant changes to the muscle fiber type suggesting atrophy in skeletal muscle of Atoh8 mutants. We further report that Atoh8 knockout mice suffer from a condition similar to ambient hypoxia which may be the primary cause of the phenotype. Altogether, this study shows the significance of Atoh8 not only in myogenesis but also in the maintenance of skeletal muscle.

## 1 Introduction

Skeletal muscles of the body originate from somites which are derived from paraxial mesoderm. Within the somites, the pre-myogenic and myogenic progenitors arise and undergo several phases of proliferation and differentiation, ultimately forming myotubes by the fusion of myocytes. In mice, myogenesis occurs sequentially in phases to give rise to distinct myofibers such as primary (embryonic), secondary (fetal) and adult myofibers while the earlier formed fibers serve as a template for the new fibers (Stockdale, 1992; Murphy and Kardon, 2011). During primary myogenesis, the Pax3+ dermomyotomal progenitor cells fuse to form embryonic myofibers. During secondary myogenesis, a subset of Pax3+ cells begin to express Pax7 by downregulating Pax3. These Pax7+ myogenic progenitor cells use embryonic myofibers as a scaffold and further fuse to form fetal myofibers (Kassar-Duchossoy et al., 2005; Hutcheson et al., 2009). A subset of these Pax7+ (satellite) cells localize themselves under the basal lamina of myofibers and serve as muscle stem cells in adult animals (Kassar-Duchossoy et al., 2005; Gokhin et al., 2008; Hutcheson et al., 2009). In the first weeks of the postnatal stage, this pool of Pax7+ cells contribute to hypertrophy of myofibers by adding new nuclei to the pre-existing fibers (White et al., 2010). In adults, hypertrophy continues but is independent of the addition of new nuclei. However, following injury or inflammation, the quiescent Pax7+ satellite cells are activated which further divides asymmetrically generating satellite cells (Pax7+/Myf5−) and myoblasts (Pax7−/Myf5+) that participate in the regeneration of the skeletal muscle (Kuang et al., 2007; Brand-Saberi and Offei, 2020).

Myogenesis is regulated by basic helix-loop-helix (bHLH) transcription factors collectively known as muscle regulatory factors (MRFs) such as Myf5, MyoD, Myogenin (MyoG) and Mrf4. Myf5, MyoD and Mrf4 govern lineage commitment and proliferation. At the same time, MyoD and Mrf4 together with MyoG also drive terminal differentiation (Bergstrom and Tapscott, 2001; Sambasivan et al., 2013; Comai and Tajbakhsh, 2014). Despite their important role in the determination and differentiation of myoblasts, the knockout mouse models of Myf5, Mrf4 and MyoD suggest that these transcription factors can compensate for each other to a certain extent (Rudnicki et al., 1992; Rudnicki et al.,1993; Bergstrom and Tapscott, 2001). However, the knockout model of MyoG resulted in embryonic lethality indicating that MyoG is essential for myofiber differentiation and organization (Hasty et al., 1993).

Despite its primary function in locomotion, skeletal muscle also plays an important role in the whole-body metabolism and the maintenance of protein homeostasis. It has been reported that during aging or in systemic disease conditions (cancer, chronic heart failure and obesity) the homeostasis of skeletal muscle is altered resulting in the loss of muscular mass. It is necessary to maintain the balance between anabolic and catabolic pathways to prevent either hypertrophy or atrophy of skeletal muscles. Early studies have identified that Akt/mTOR signaling plays a key role in skeletal muscle growth, its activation was shown to be associated with hypertrophy (Bodine et al., 2001). Akt/mTOR signaling has been further shown to be a master regulator of protein synthesis which is activated by upstream factors such as insulin, IGF1, amino acids and mechanical strain. As it is crucial for muscle growth, its dysregulation is associated with increased protein degradation and decreased protein synthesis (Laplante and Sabatini, 2012).

Atoh8 is a member of the bHLH family of transcription factors which has been shown to play an important role in organogenesis, maintenance of pluripotency and cancer (Divvela et al., 2022). Unlike muscle regulatory factors that are expressed only in myogenic cells, Atoh8 is ubiquitously expressed during embryonic and adult life. In our previous study that was performed on chicken embryos, we have identified that Atoh8 is expressed in the hypaxial myotome of somites and silencing Atoh8 in the ventrolateral lip of the avian dermomyotome resulted in the disruption of hypaxial myotome formation suggesting that Atoh8 participates in specific aspects of skeletal muscle differentiation (Balakrishnan-Renuka et al., 2013). Following this, in our recent study, we have identified the expression of Atoh8 in satellite cells and proliferating myoblasts of regenerating myofibers, at the same time its co-localization with Pax7, vimentin, nestin and neonatal myosin heavy chain suggests that Atoh8 is activated during myoblast proliferation with a drastic decrease during differentiation. Studies on human patients revealed that Atoh8 was also observed to be highest in regenerating myofibers in inflammatory myopathies and muscular dystrophy compared to healthy controls (Güttsches et al., 2015).

In the current study, we evaluated the effects of loss of Atoh8 on adult myogenesis and skeletal muscle regeneration in mice. We show for the first time that Atoh8 regulates myoblast proliferation and that its upregulation delays the onset of differentiation. We also identified changes in the skeletal muscle fiber constitution in Atoh8 knockout (KO) mice, which correlates with the phenotype of mice suffering from ambient hypoxia. In addition to this, we identified that Atoh8 knockout mice suffer from atrophy potentially induced by hypoxia. Based on our data we propose that Atoh8 regulates myogenesis via Akt/mTOR signaling mediated by myostatin.

## 2 Materials and methods

### 2.1 Isolation of mouse primary myoblasts

Due to lack of highly specific Anti-Atoh8 antibody we used *Atoh8* ^*Flag-tag*^ mice as a control. Mouse primary myoblasts were isolated from adult wildtype (*Atoh8* ^*Flag-tag*^ *or WT*) and Atoh8 knockout (Atoh8^−/−^ or KO) C57Bl/6NJ mice as described by (Hindi et al., 2017). Mouse primary myoblasts were maintained in DMEM high glucose (Thermo Fisher Scientific) supplemented with 20% FBS (Pan-Biotech), 1% L-Glutamine (Life technologies), 1% NEAA (Life Technologies), 1% Penicillin-streptomycin (Life Technologies) and 10 µg/ml bFGF (Peprotech). Differentiation was induced once the myoblasts were 70%–80% confluent using differentiation media containing DMEM high glucose (Thermo Fisher Scientific), 2% horse serum (Pan-Biotech) and 1% Penicillin-streptomycin (Life Technologies). Differentiation was performed on 2% Matrigel (Corning, Amsterdam, Netherlands) coated dishes.

### 2.2 Generation of atonal homolog 8-flag overexpressing C2C12 cells

We have modified the commercially available immortalized mouse myoblast cell line (C2C12) to overexpress Atoh8-Flag sequence. A stable Atoh8-Flag overexpression in C2C12 cells was achieved by transducing C2C12 cells with retrovirus carrying the Atoh8-Flag sequence (C2C12-OE). Virus production was performed using HEK cells as previously described (Divvela et al., 2019). The C2C12 cells (5 × 10^5^) were infected with 50 µL of virus twice on consecutive days with an incubation time of 24 h. Actual experiments were performed at least two passages after the infection. Differentiation was induced once the C2C12 and C2C12-OE myoblasts were 70%–80% confluent using differentiation media containing DMEM high glucose (Thermo Fisher Scientific), 2% horse serum (Pan-Biotech) and 1% Penicillin-streptomycin (Life Technologies). Differentiation was performed on 2% Matrigel (Corning, Amsterdam, Netherlands) coated dishes.

### 2.3 BrdU staining

BrdU staining is an approach to determine how fast cells of a certain population proliferate. This technique is based on the addition of BrdU, a modified synthetic nucleotide, to the growth media of the cells. This thymidine analog is incorporated into the cells during DNA replication in the positions where thymidine should be present. Cells that divide during the considered exposure time incorporate it into their nuclei, whereas cells that do not divide during this time lack BrdU in their nuclei. To perform this experiment, 10 μM of BrdU (5-Bromo-2´-Deoxyuridine) #B23151 (Invitrogen™) was added to growth media and cells were kept in these conditions for 10 h in the case of primary myoblasts and 3 h in the case of C2C12. Following the incubation time, the media was removed from the dishes and cells were washed three times with PBS. Cells were fixed with 4% PFA/PBS for 15 min at room temperature and washed again with PBS. Cells were then permeabilized by the addition of 1% Triton X-100 (Thermo Fisher) and incubated for 20 min at room temperature. DNA was hydrolyzed by treating cells with 1N HCl (on ice) and 2N HCl (room temperature) each with 10 min of incubation. The reaction was stopped by removal of the acid and addition of phosphate/citrate buffer (pH 7.4) with incubation for 10 min at room temperature. Detection of BrdU + nuclei was achieved by performing immunostaining using Anti-BrdU antibody. Images were taken using Zeiss LSM 800 and analyzed using ImageJ.

### 2.4 Cardiotoxin induced skeletal muscle injury and regeneration of WT and KO mice

To assess the competence of skeletal muscle regeneration in WT (*Atoh8* ^*Flag-tag*^) and KO (*Atoh8* ^*−/−*^) mice, we subjected 3 mice per time point for each genotype (2 males and one female) between 3–6 months of age to cardiotoxin-induced injury and quantified the regeneration process by measuring the cross-sectional area and by sampling the size distribution of myofibers on day 5, 10 and 14 and 30 µL of 10 µM cardiotoxin (CTX) was injected into the right tibialis anterior (TA) muscle. The left TA muscle was injected with 1xPBS which served as non-injured control. The cardiotoxin was injected after the mice were anesthetized with isoflurane (5% v/v). The TA muscle tissues were harvested on specific days following cardiotoxin injection on days 3, 5, 10 and 14 and fixed with 4% paraformaldehyde. The fixed tissues were sectioned and stained with hematoxylin and eosin and imaged. The obtained images were quantified for cross-sectional area (CSA) using ImageJ. Mice were bred in-house and housed on a 12 h dark/light cycle with food and water *ad libitum*. The study was carried out in accordance with the European Communities Council Directive of 2010 (2010/63/EU) for the care of laboratory animals and approved by the animal care committee of North Rhine-Westphalia, Germany, based at the LANUV (Landesamt für Umweltschutz, Naturschutz und Verbraucherschutz. Nordrhein-Westfalen, D-45659, Recklinghausen, Germany).

### 2.5 Spontaneous differentiation of murine induced pluripotent stem cells

Murine induced pluripotent stem cells (miPSCs) used in this study were cultured in 2i-LIF media as described previously in (Divvela et al., 2019). The cultured miPSC colonies were dissociated with the TRYPLE reagent (Life Technologies). The resuspended cells were cultured in droplets of 20 µL with a density of 100 cells/µL and hung off the lid of a culture dish for 48 h in 15% KOSR media (Life Technologies). Following the formation of embryoid bodies (EBs), 30 embryoid bodies were transferred to 2% Matrigel (Corning, Amsterdam, Netherlands) coated 35 mm dishes and further cultured for 21 days. RNA was isolated on Day 0 (48-h EBs), Day 7, 14 and 21 and mRNA expression of myogenic markers were analyzed. Media was changed every alternate day during differentiation.

### 2.6 Directed differentiation of murine embryonic stem cells

Murine embryonic stem cells (mESCs) used in this study were cultured in 2i-LIF media as described previously in (Divvela et al., 2019). Directed differentiation of WT- and KO-ESCs was performed according to the serum-free protocol described by (Chal et al., 2015). However, the seeding density for ESCs was optimized to 5,000 cells/cm^2^ for better results.

### 2.7 RNA isolation, reverse transcription and real-time PCR

RNA was isolated using TRI reagent (Sigma-Aldrich). 1 µg of RNA was used to perform a reverse transcriptase reaction using GoScript Reverse transcriptase (Promega, Mannheim, Germany). GoTaq qPCR master mix (Promega) was used for Real-time quantitative PCR reaction. These reactions were performed by following the respective manufacturer’s instructions. The Livak method (Livak and Schmittgen, 2001) was used to calculate relative quantification. The list of Real-Time qPCR primers used in the study are available in the supplementary information.

### 2.8 Immunostaining

Cells grown on 4-well plates were washed thrice with phosphate-buffered saline 1xPBS (Thermo Fisher Scientific), followed by fixation with 4% paraformaldehyde (VWR) in 1xPBS for 15 min. The cells were then permeabilized with 0.5% Triton X-100 (Merck) for 15 min and blocked with 5% Bovine Serum Albumin, BSA (Life Technologies) for 30 min at room temperature. Following blocking, cells were incubated with primary antibodies at 4°C overnight after which the cells were washed thrice with 1xPBS and incubated with respective secondary antibodies for 1 h. Following this, the cells were washed thrice with 1xPBS and mounted using mounting media containing DAPI (Invitrogen, S36920). Antibodies used in the immunostaining are listed in supplementary information.

### 2.9 Western blot

The Trizol protein isolation method was used to extract proteins from cells grown on 6-well plates. Isolated proteins were quantified using Bradford’s assay (Sigma Aldrich, BCA1-1KT). 50 µg of proteins were loaded on 12.5% SDS-Page gel. Western blot was performed according to Abcam Western blot protocol. Following blotting, the blots were incubated with respective antibodies at 4°C overnight after which the blots were incubated with respective secondary antibodies conjugated with HRP for 1 h at room temperature. ECL reagent (Biorad, 1705060) was used for imaging. Antibodies used in the Western blot are listed in Supplementary Material.S1.

### 2.10 Periodic acid-schiff staining

The skeletal muscle tissues were snap-frozen in OCT media using liquid nitrogen. The tissues were sectioned at a thickness of 7 um using a cryostat. The slides were then stained with periodic acid-Schiff (PAS) according to the manufacturer’s protocol (Carl Roth GmbH) to detect glycogen in muscle structure. The slides were first rehydrated with distilled water and treated with 1% periodic acid for 10 min. The slides were then rinsed well under running tap water (10 min) and distilled water twice for 2 mins each. After this, the slides were covered with Schiff’s reagent and incubated for 15 min. Following this, the slides were washed under running tap water for 10 min. The slides were then counterstained with Haematoxylin solution for 5 min followed by a wash under running tap water (10 min). The slides were rinsed in increasing concentrations of alcohol (70%, 80%, 95%, and 100%) and mounted using mounting media. The images were captured using Zeiss Axioscan 7 at × 20 magnification. The myofiber type was quantified using ImageJ based on the glycogen content. The higher the glycogen, the darker the fiber.

### 2.11 Behavioral analysis

A battery of behavioral tests including the gait analysis, rotarod, vertical pole test, hang wire and beam walk were performed to assess the motor coordination of WT (*Atoh8* ^*Flag-tag*^) and KO (*Atoh8* ^*−/−*^) mice. Five males and four females at 3 months of age from each group were used. Mice were bred in-house and housed on a 12 h dark/light cycle with food and water *ad libitum*. The study was carried out in accordance with the European Communities Council Directive of 2010 (2010/63/EU) for care of laboratory animals and approved by the animal care committee of North Rhine-Westphalia, Germany, based at the LANUV (Landesamt für Umweltschutz, Naturschutz und Verbraucherschutz. Nordrhein-Westfalen, D-45659, Recklinghausen, Germany). The study was supervised by the animal welfare commission of the Ruhr-University Bochum. All efforts were made to minimize the number of mice used. All experiments were conducted during the wake cycle of the mice. The average of all trials is presented as mean ± SEM (standard error of the mean). The statistical significance was calculated using the Holm-Sidak method using Graphpad. Statistical significance is shown a*s* *p ≤ 0.05, **p ≤ 0.01, and ***p ≤ 0.001.

#### 2.11.1 Gait analysis

For gait analysis, the paws of mice were painted with non-toxic, water-soluble children’s paint (Pelikan). The forepaws were painted red and the hind paws blue. To analyze footprints, mice were placed at one end of a 10 cm wide × 70 cm long × 10 cm high tunnel which was connected to their corresponding home cages. Mice were trained to walk at an even pace to the end of the tunnel before their footprints were taken. A total of 7 steps were measured for each mouse which was further scanned into a computer and analyzed by ImageJ software (NIH) as described previously (Klapdor et al., 1997). Stride length and paw width were measured for 42 steps per group.

#### 2.11.2 Rotarod test

The rotarod test was used to assess the motor coordination of WT and KO mice. Mice were habituated for 1 min on the rotarod at a speed of 4 rpm. Then the speed was accelerated from 4 to 40 rpm at 0.1 rpm/s until the mice fell off the rotarod. For the endurance test, the speed was set to 10 rpm, and the duration of the mice remained on the rod was measured. The latency to fall (s) and speed of rod (rpm) at the time of fall were recorded for each mouse. Each mouse performed 3 trials.

#### 2.11.3 Vertical pole test

The vertical pole test was performed to assess the motor coordination and balancing abilities of the mice. A grooved metal pole (50 cm long × 1 cm wide) was secured to a stable platform. Mice were placed face upwards on the top of the pole and the latency to reorient and climb down the pole was recorded. A maximum of 120 s was given if the mouse did not complete the test within 120 s or if the mouse fell off the pole. Each mouse performed one trial.

#### 2.11.4 Hang wire test

A hanging wire test was performed to evaluate the balance capabilities and muscular strength of the mice. In this test, mice were hung upside down on a wire grid (12 mm × 12 mm) positioned 50 cm above the cage. The latency to fall was recorded with a maximum score of 60 s. The mice that did not fall after 60 s were also given a maximum score.

#### 2.11.5 Beam walk test

A beam walk test was performed to assess fine motor coordination skills and balancing capabilities. Mice were placed on a 10 mm wide × 70 cm long elevated beam. One end of the beam is mounted to a small illuminated platform and the opposite end to a 20 cm^2^ goal box. Mice were trained for 2 days (6 trials per day) to walk to the goal box. On the third day, the time each mouse took to reach the goal box, the time mice spent idling (immobile) at the start position on the beam and the number of left and right hindpaw slips were recorded. A maximum of 120 s was given for the mice to reach the goal box. Falls from the beam were recorded as 120 s. Each mouse performed 3 trials. Data were presented as the average of 3 replicates.

## 3 Results

### 3.1 Loss of atonal homolog 8 results in reduced proliferation and enhanced differentiation of myoblasts

**FIGURE 1:**
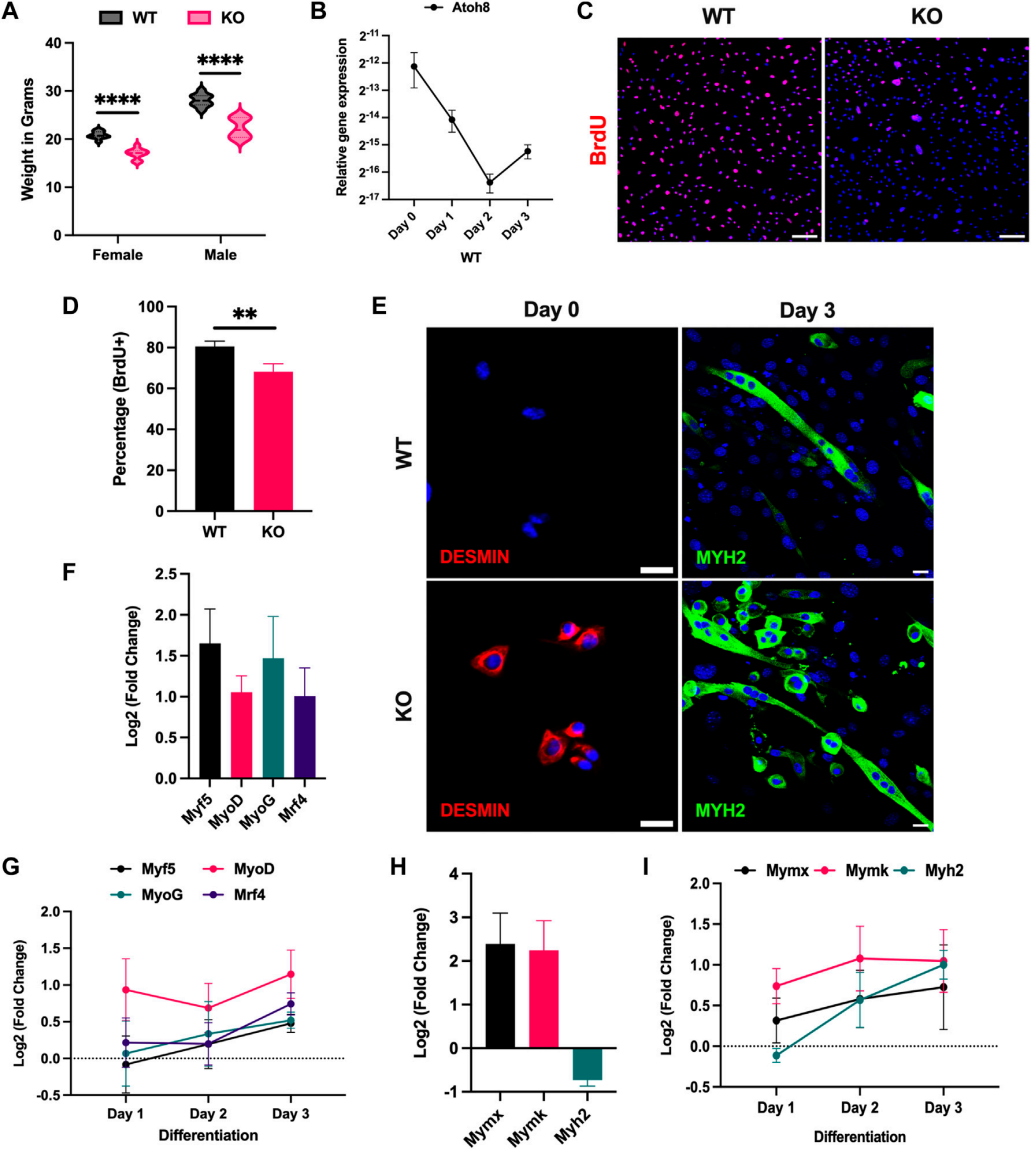
Atoh8 regulates myoblast proliferation and differentiation. **(A)** Comparison of the body weight of 3 months old male and female mice belonging to both genotypes WT and KO in grams. **(B)** mRNA expression of Atoh8 in the proliferative phase (Day 0) and during days 1, 2, and 3 of differentiation. **(C)** Representative images of WT and KO primary myoblasts stained with Anti-BrdU antibody following 10 hours of incubation with 10 μM BrdU. Nuclei were counterstained with DAPI. The data are shown as the percentage of the ratio of the number of BrdU cells to the total number of cells. The scale bar represents 100 μm. **(D)** Graph depicting the percentage of BrdU positive cells in WT and KO primary myoblasts following treatment with 10 μM BrdU for 10 hours. **(E)** Representative pictures showing immunostaining of primary myoblasts (WT and KO) with Desmin on Day 0 (high serum and proliferative phase) and Myosin heavy chain 2 (MYH2) on Day 3 during differentiation (low serum). The scale bar represents 20 μm. Nuclei were counterstained with DAPI. **(F)** Expression of muscle regulatory factors (Myf5, MyoD, MyoG and Mrf4) in KO primary myoblasts relative to WT (proliferative phase). **(G)** Expression of muscle regulatory factors (Myf5, MyoD, MyoG and Mrf4) in KO primary myoblasts relative to WT (differentiation). **(H)** Expression of myoblast fusion markers (Mymx and Mymk) and differentiation marker (Myh2) in KO primary myoblasts relative to WT (proliferative phase). **(I)** Expression of myoblast fusion markers (Mymx and Mymk) and differentiation marker (Myh2) in KO primary myoblasts relative to WT (differentiation). The gene expression shown in this figure is normalized to the 18s. The statistical significance was calculated using the Holm-Sidak method using Graphpad. Statistical significance is shown as follows no significance *p* > 0.05, *p ≤ 0.05, **p ≤ 0.01, ***p ≤ 0.001, and ****p ≤ 0.0001. The data shown in the figure is the mean ± SEM of 3 replicates. Loss of Atoh8 alters fiber type composition in mouse skeletal muscle.

In comparison to WT mice (*Atoh8* ^*Flag-tag*^ mice in which the Atoh8 gene is tagged with 3x Flag-sequences), the KO (*Atoh8* ^*−/−*^) mice showed a significantly reduced body weight. Since skeletal muscle accounts for up to 40% of the body mass and Atoh8 is a bHLH transcription factor expressed in muscle forming tissue, we were interested to understand its relevance in skeletal muscle differentiation (Figure 1A). To determine the role of Atoh8 during adult myogenesis *in vitro*, the expression of *Atoh8* was quantified at the mRNA level in primary myoblasts as well as during their differentiation. We observed a higher expression of *Atoh8* in myoblasts during the proliferative phase (under growth conditions) compared to the time points assessed during differentiation. A sharp decline in the expression of *Atoh8* was observed following induction of differentiation from Day 0 to Day 2, however, on Day 3 the expression of *Atoh8* was observed to increase again. This data suggests that Atoh8 has a function during the proliferative phase, whereas it is not required during the transition of myoblasts from the proliferative to the differentiation phase. However, subject to its upregulation again on day 3, Atoh8 once again seemed to be involved in differentiation (Figure 1B). First, to comprehend if Atoh8 has any functional role in the regulation of proliferation, we subjected primary myoblasts derived from WT and KO to BrdU proliferation assay. The BrdU assay indicated a significantly lower proliferation in KO myoblasts compared to WT (Figures 1C,D). At the same time, KO primary myoblasts were observed to fuse prematurely under growth conditions (high serum). Next, to evaluate the impact of the loss of Atoh8 on myogenic differentiation, the primary myoblasts of WT and KO were subjected to differentiation by changing culture conditions from high serum (20%) to low serum (2%). Upon serum reduction, KO primary myoblasts showed an enhanced differentiation process compared to WT, this was further confirmed by immunostaining against Desmin on Day 0 (proliferative phase) and MYH2 (myosin heavy chain 2) on day 3 of differentiation (Supplementary Figure S1; Figure 1E) and by fusion index (Supplementary Figure S2A).

Analysis of muscle regulatory factors (MRFs) such as *Myf5, MyoD, MyoG,* and *Mrf4* revealed that these factors are upregulated in KO myoblasts in the proliferative phase as well as during differentiation compared to the myoblasts derived from WT (Figures 1F,G). In addition to this, we have also analyzed differentiation markers involved in myoblast fusion and myotube formation such as *Myomixer (Mymx)*, *Myomaker (Mymk),* and *Myh2*. The KO myoblasts which are maintained in high serum media in the proliferative phase showed a premature upregulation of *Mymx* and *Mymk.* However, a slight downregulation of *Myh2* was observed at this timepoint in KO myoblasts compared to WT (Figure 1H). Additionally, these markers were also evaluated during differentiation (with 24 h interval for 72 h). As expected, *Mymx* and *Mymk* were found to be upregulated in differentiating KO myoblasts compared to WT. In contrast to our observation in the proliferative phase, from Day 2 of differentiation, the expression of *Myh2* was observed to be upregulated in KO differentiating myoblasts (Figure 1I). Overall, this data indicates that Atoh8 positively regulates myoblast proliferation and negatively regulates myogenic differentiation.

### 3.2 Loss of Atoh8 alters fiber type composition in mouse skeletal muscle

**FIGURE 2:**
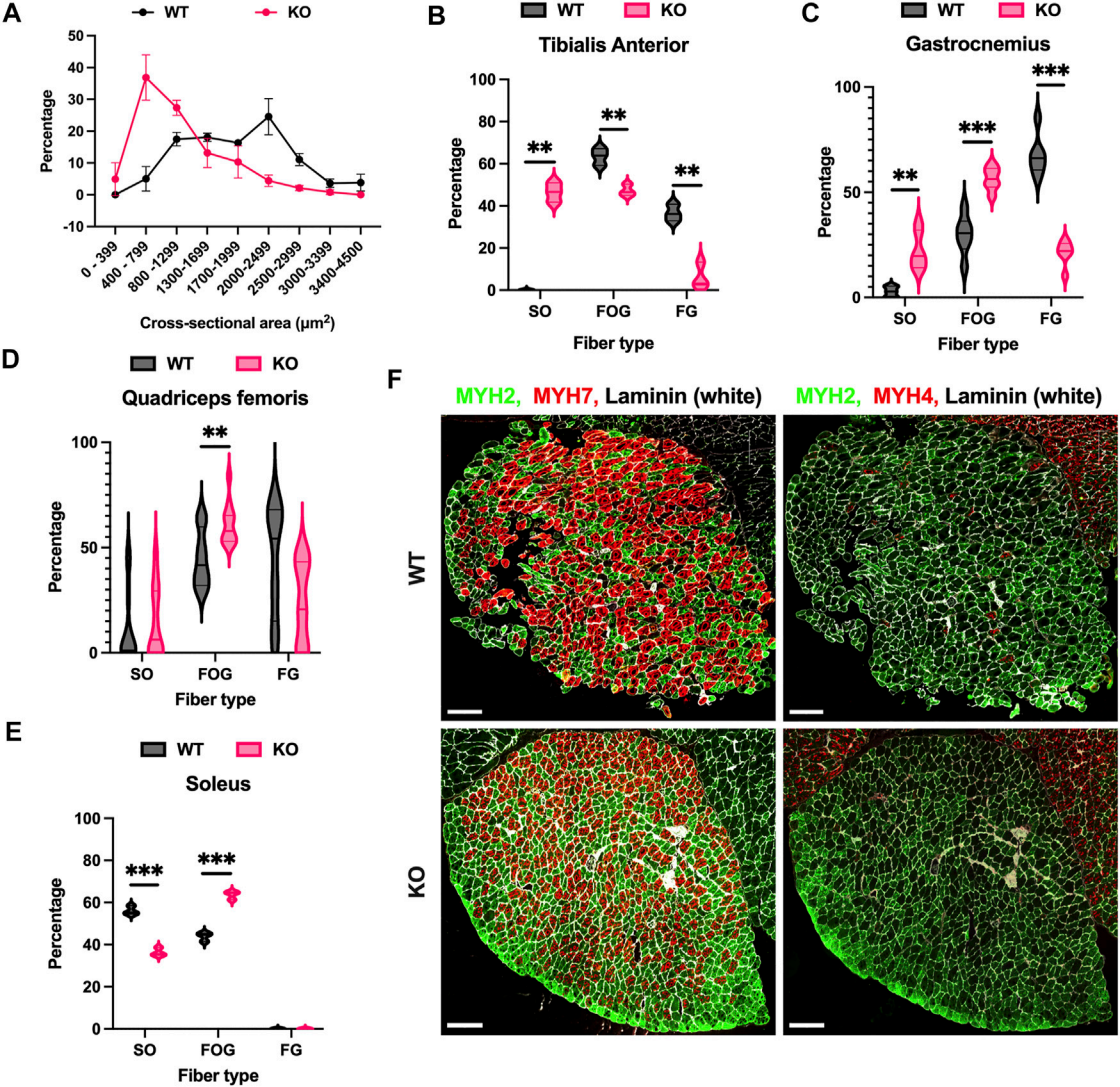
Loss of Atoh8 affects myofiber size and type. **(A)** Graph shows the cross-sectional area of the myofibers distributed in the tibialis anterior muscle. The KO mice have predominantly thin myofibers compared to WT mice. **(B–D)** The violin plots show myofiber distribution in the Tibialis Anterior **(B)**, Gastrocnemius **(C)**, and Quadriceps femoris **(D)**. SO represents (slow oxidative), FOG (fast oxidative and glycolytic) and FG (fast glycolytic). **(E)** The violin plot shows myofiber distribution in the soleus muscle. **(F)** Representative images of immunostaining performed on soleus muscle once again confirm the higher number of MYH2 positive fibers (FOG) compared to MYH7 positive fibers (SO) in KO compared to WT (Left). Representative images of immunostaining performed on soleus muscle show a higher number of MYH2 positive fibers (FOG) compared to MYH4 positive fibers (FG) (Right). The scale bar represents 200 μm. The statistical significance was calculated using the Holm-Sidak method using Graphpad. Statistical significance is shown as follows no significance *p* > 0.05, *p ≤ 0.05, **p ≤ 0.01, and ***p ≤ 0.001. The data shown in the figure is the mean ± SEM of 3 replicates.

Following *in vitro* comparison of WT and KO myoblasts, we further analyzed skeletal muscle tissue. We first examined the thickness of the muscle fibers by measuring the cross-sectional area of each of the individual fibers in “tibialis anterior muscle” isolated from WT and KO mice. Interestingly, the majority (36.87%) of myofibers in KO mice were found to be thinner (400–700 µM^2^) compared to the majority (24.55%) of myofibers in WT mice (2000–2,499 µM^2^). The comparison of the thickness of myofibers between WT and KO tissues is shown in (Figure 2A) as size distribution based on cross-sectional area. Subsequently, we performed PAS staining to analyze the muscle fiber type composition of different muscles of the lower limb in both WT and KO mice. To our surprise, we observed a substantial increase in the number of slow oxidative (SO) muscle fibers in KO animals compared to controls (WT). Next, we quantified the number of fast oxidative glycolytic (FOG) and fast glycolytic (FG) fibers in the muscle tissue using PAS-staining. We observed that the increase in the number of slow oxidative fibers in KO is because of the shift from fast muscle fiber type, specifically from the fast glycolytic type (Figures 2B–D; Supplementary Figure S3). However, as an exception, soleus muscle which is normally comprised of a high number of slow oxidative fibers was observed to have more fast oxidative fibers in KO compared to control (WT) with a difference of 20% (Figure 2E). To substantiate the disparity in the muscle fiber composition of the soleus muscle, we further performed immunostaining of the soleus muscle with antibodies against slow oxidative fibers (MYH7), fast oxidative (MYH2) and fast glycolytic (MYH4) fibers. Consistent with PAS staining, the immunostaining of the soleus muscle also showed a significant increase in the number of fast oxidative fibers (FOG) (Figure 2F). Altogether, the data revealed that the loss of Atoh8 results in significant changes to the myofiber size and fiber type.

### 3.3 Overexpression of atonal homolog 8 promotes myoblast proliferation but perturbs differentiation

**FIGURE 3:**
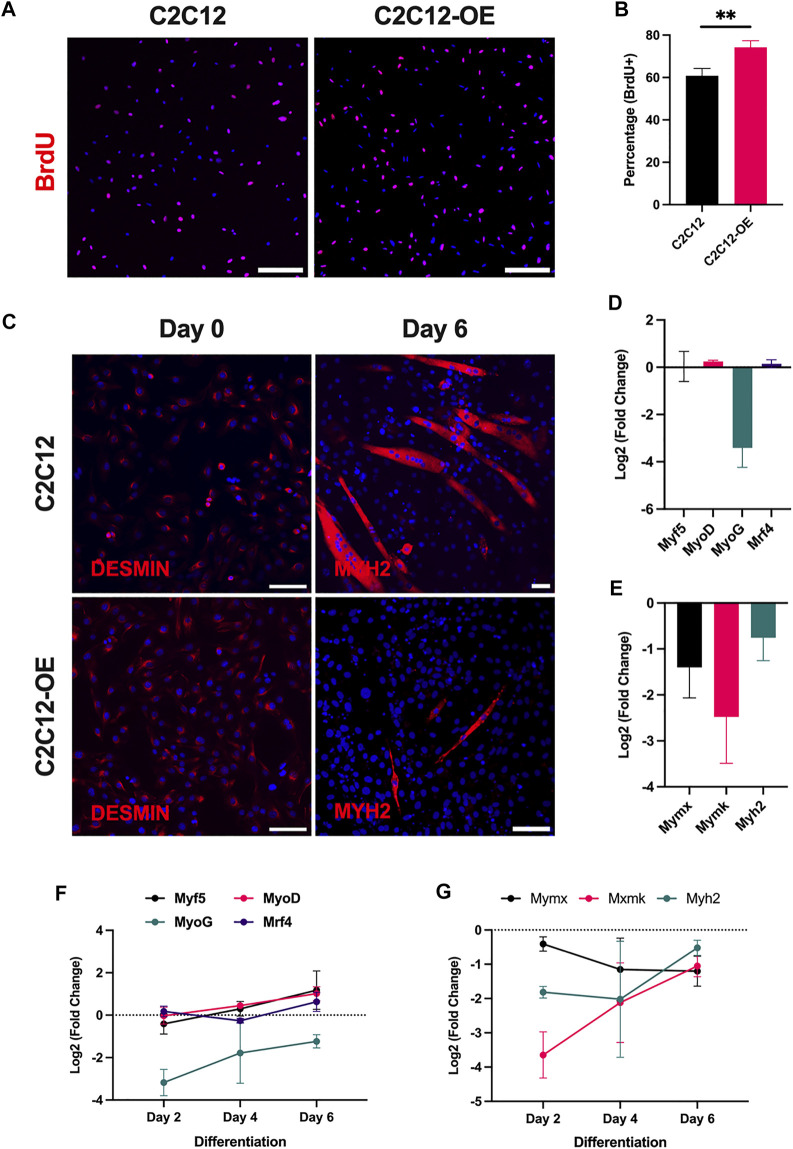
Atoh8 overexpression promotes proliferation but perturbs differentiation. **(A)** Representative images of C2C12 and C2C12-OE myoblasts stained with Anti-BrdU antibody following 3 hours of incubation with 10 μM BrdU. Nuclei were counterstained with DAPI. The scale bar represents 100 μm. **(B)** Graph depicting the percentage of BrdU positive cells in C2C12 and C2C12-OE myoblasts following treatment with 10 μM BrdU for 3 hours. The proliferative rate in C2C12-OE myoblasts was found to be significantly higher compared to C2C12 (control). **(C)** Representative pictures showing immunostaining of myoblasts (C2C12 and C2C12-OE) on Day 0 (high serum and proliferative phase) and myotubes on Day 6 of differentiation (low serum). Both C2C12 and C2C12-OE myoblasts stained positive for Desmin (Left) in the proliferative phase. The immunostaining of myotubes formed as a consequence of differentiation of C2C12 and C2C12-OE on Day 6 stained positive for MYH2. Nuclei were counterstained with DAPI. The scale bar represents 50 μm. **(D)** Expression of muscle regulatory factors (Myf5, MyoD, MyoG and Mrf4) in C2C12-OE myoblasts. The data shown are normalized to 18s and relative to C2C12 cells (proliferative phase). **(E)** Expression of myoblast fusion markers (Mymx and Mymk) and differentiation marker (Myh2) in C2C12-OE myoblasts. The data shown are normalized to 18s and relative to C2C12 cells (proliferative phase). **(F)** Expression of muscle regulatory factors (Myf5, MyoD, MyoG and Mrf4) in C2C12-OE myoblasts during days 2, 4 and 6 of differentiation. The data shown are normalized to 18s and relative to C2C12 cells (Control). **(G)** Expression of myoblast fusion markers (Mymx and Mymk) and differentiation marker (Myh2) in C2C12-OE myoblasts. The data shown are normalized to 18s and relative to C2C12 cells. The statistical significance was calculated using the Holm-Sidak method using Graphpad. Statistical significance is shown as follows no significance *p* > 0.05, *p ≤ 0.05, **p ≤ 0.01 and ***p ≤ 0.001. The data shown in the figure is the mean ± SEM of 3 replicates.

Given the impact of the loss of Atoh8 on myogenesis as shown previously, we next investigated how over-expression of Atoh8 affects myoblast proliferation and differentiation. To answer this, we generated a C2C12 myoblast cell line where Atoh8 along with a 3x Flag-sequence tag was stably expressed. The stable Atoh8 overexpression was achieved by transducing the C2C12 myoblast cell line with a retrovirus carrying the Atoh8-Flag sequence. Following this, a BrdU proliferation assay was performed to check if Atoh8 overexpression can influence myoblast proliferation. As expected C2C12-OE (Atoh8-Flag overexpressing) cells were found to proliferate at a higher rate compared to the control (C2C12) cells (Figures 3A,B). To make sure that this effect is not from the random retroviral integration, we generated multiple Atoh8-Flag overexpression cell lines and all of them showed higher rates of proliferation compared to control cells (data not shown). Subsequently, C2C12 and C2C12-OE lines were subjected to differentiation. As anticipated, a delayed differentiation of myoblasts was observed in C2C12-OE cells compared to control (C2C12). On day 6, following the induction of differentiation, the cells were fixed and immunostained against MYH2 to determine the myogenic fusion index. We observed a significantly lower myogenic index in C2C12-OE cells compared to control (C2C12) cells suggesting a poor differentiation potential of these cells following Atoh8 overexpression (Figure 3C; Supplementary Figure S2B). We further quantified the expression of MRFs at mRNA level such as *Myf5, MyoD, MyoG,* and *Mrf4* during proliferation and differentiation. In the proliferative phase (Day 0), no difference in the expression of *Myf5, MyoD,* and *Mrf4* was observed. However, we detected a strong downregulation of *MyoG* (3.41-fold) in the C2C12-OE myoblasts compared to control (C2C12) cells. During differentiation, we did not detect any major change in the expression of *Myf5, MyoD,* and *Mrf4.* Nevertheless, *MyoG* which plays a major role in the terminal differentiation of myoblasts was found to be consistently downregulated in C2C12-OE cells compared to the control cells (Figures 3D,F). Following this, we analyzed myoblast fusogenic markers (*Mymx and Mymk*) and differentiation marker (*Myh2*). Compared to the control (C2C12), C2C12-OE cells showed a consistent downregulation of *Mymx, Mymk, and Myh2* during proliferation and throughout differentiation (Figures 3E,G). Altogether, this data once again underlines the regulatory effect of Atoh8 on myoblast proliferation and skeletal myogenesis.

### 3.4 Loss of atonal homolog 8 results in premature differentiation following cardiotoxin induced skeletal muscle injury and subsequent regeneration

**FIGURE 4:**
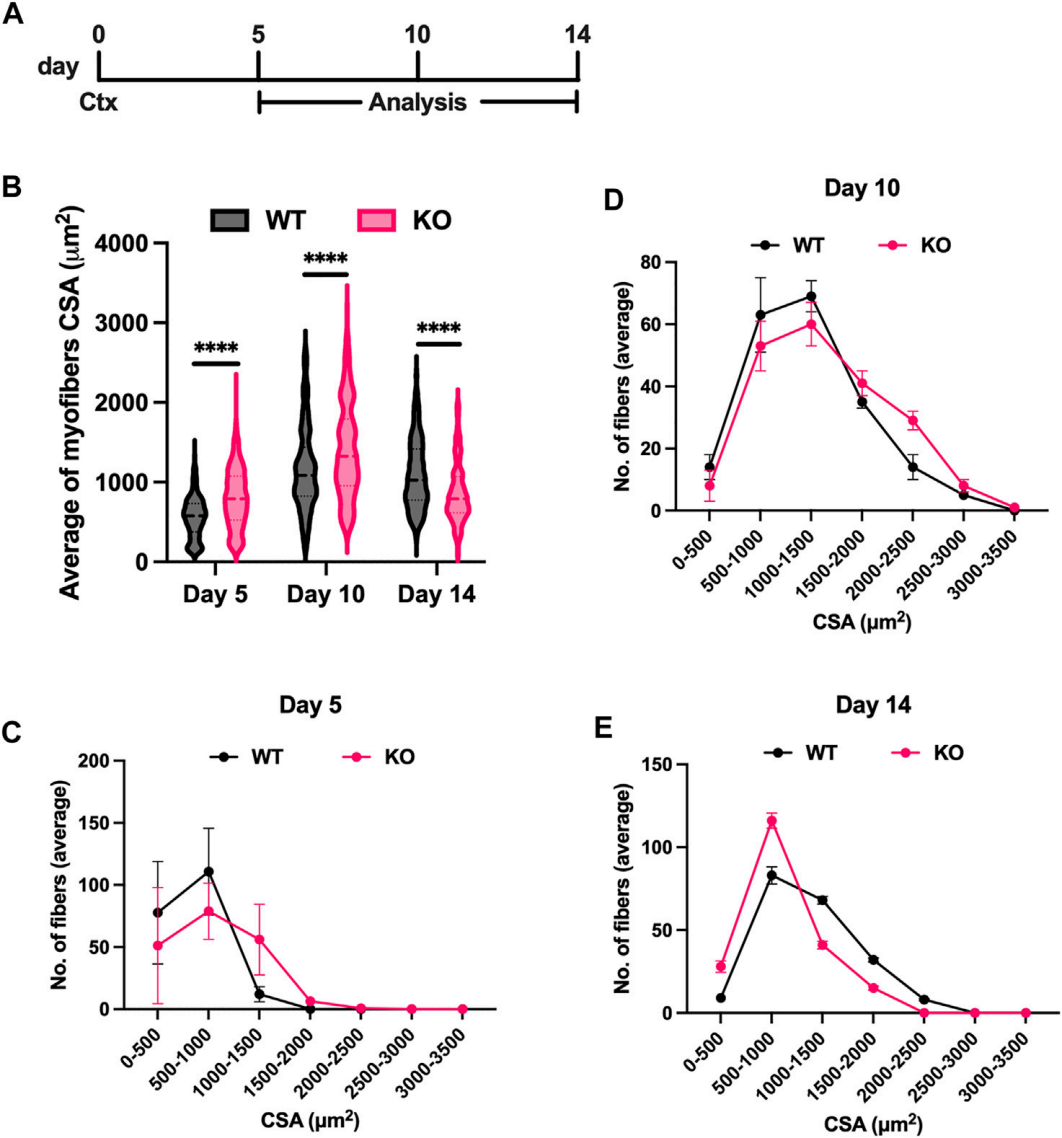
Loss of Atoh8 results in premature onset of differentiation during skeletal muscle regeneration. **(A)** Experimental plan of acute cardiotoxin (Ctx) induced skeletal muscle injury. **(B)** Average cross-sectional area (CSA) of centrally nucleated myofibers on days 5, 10 and 14 after injury. **(D, E)** Size distribution of myofibers based on the cross-sectional area on days 5, 10 and 14 after injury. Statistical significance is shown as no significance *p* > 0.05, *p ≤ 0.05, **p ≤ 0.01, ***p ≤ 0.001 and ****p ≤ 0.0001. The values shown are mean ± SEM, 3 mice per group. A total of 512 fibers were analyzed for each time point.

To further understand the significance of Atoh8 during regeneration, we assessed the competence of regeneration in WT and KO mice following cardiotoxin induced tibialis anterior (TA) muscle injury (Figure 4A). Analysis performed after 72 h of injury showed successful infiltration of inflammatory cells in WT and KO tissues. By day 5, we could observe centrally nucleated regenerating myofibers both in WT and KO mice. Further analysis of the cross-sectional area of centrally nucleated myofibers showed thick myofibers in KO mice on day 5 (801.40 ± 16.41) and day 10 (1,401.98 ± 26.56) compared to day 5 (563.12 ± 11.45) and day 10 (1,166.73 ± 23.20) in WT mice. In contrast to the findings observed on day 5 and 10, the cross-sectional area of myofibers on day 14 (861.23 ± 16.50) revealed thin myofibers in KO mice compared to WT (1,095.67 ± 19.02) suggesting premature onset of differentiation of KO tissues compared to WT (Figure 4B; Supplementary Figure S7). To further confirm this, we have also checked the size distribution of centrally nucleated myofibers at different timepoints during the course of regeneration. The size distribution analysis also showed higher number of thick myofibers in KO on initial timepoints (day 5 and 10), whereas on day 14, WT tissues showed a greater number of thick fibers compared to KO tissues (Figures 4C–E). The comparison of cross-sectional area and size distribution in KO and WT among initial timepoints (day 5 and 10) and the later timepoint (day 14) suggests that KO skeletal myofibers have suffered from premature differentiation as opposed to WT which seems to have thick myofibers suggesting a sufficient rate of proliferation before the onset of differentiation. The muscles in both WT and KO were observed to have largely regenerated although there was still an abundance of centrally nucleated myofibers. Together, this data further complements the observations seen *in vitro* and also confirms the significance of Atoh8 in myoblast proliferation during skeletal myogenesis and regeneration.

### 3.5 Pluripotent stem cells showed enhanced skeletal muscle differentiation in the absence of atonal homolog 8

In the view of previous observations on how the loss of Atoh8 affects myoblast proliferation and differentiation, and the phenotypes seen in KO mice such as lower muscle mass and changes in the fiber-type composition, we further questioned how the loss of Atoh8 affects pre-myogenic and myogenic development. Recently, we have identified that the loss of Atoh8 primes the pluripotent cells towards the mesendodermal phenotype *in vitro*. Early *in vitro* differentiation of KO-ESCs showed upregulation of the mesodermal marker (Brachyury), and the pre-somitic progenitor cell marker (Tbx6) in the KO compared to WT-ESCs (Divvela et al., 2019). In addition to this, our group has previously shown the expression of Atoh8 in a subset of embryonic muscle precursor cells (hypaxial myotome) in the somites. Moreover, loss of function studies performed on the myotomal compartment in chicken embryos resulted in the disruption of differentiation suggesting that Atoh8 has a function in the aspect of skeletal muscle differentiation (Balakrishnan-Renuka et al., 2013). Overall, to identify the *in vitro* differentiation potential of KO cells over longer time peroids, particularly towards the myogenic fate, we subjected iPSCs and ESCs to spontaneous and directed differentiation respectively. Directed differentiation of ESCs towards myogenic fate was performed using a serum-free protocol (Chal et al., 2015).

**FIGURE 5:**
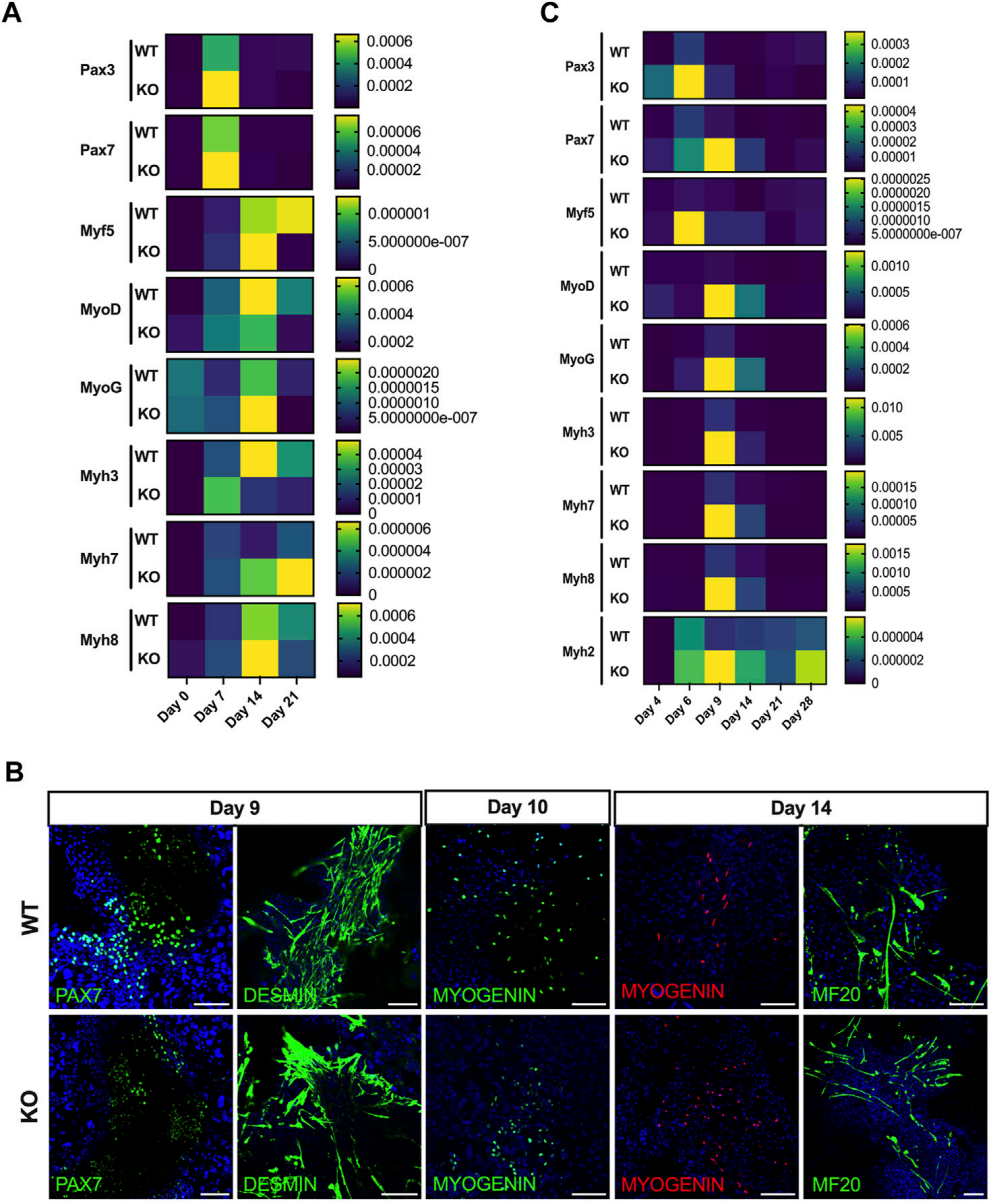
Loss of Atoh8 promotes the differentiation of pluripotent stem cells towards myogenic fate. **(A)** Expression of myogenic markers during different days (0, 7, 14, and 21) of differentiation of embryoid bodies derived from WT- and KO-iPSCs. The expression profile shows sequential activation of skeletal muscle progenitor markers (*Pax3* and *Pax7*), MRFs (*Myf5*, *MyoD*, *MyoG*) and differentiation markers (*Myh3, Myh7, Myh8*). **(B)** Representative images of cells during directed differentiation of ESCs towards myogenic fate. The upper and lower panels show the cell population stained positive for PAX7 (Day 9), Desmin (Day 9), Myogenin (Day 10 and 14) and MF20 (Day 14) during differentiation. **(C)** Expression of myogenic markers during different days (4, 6, 9, 14, 21 and 28) of differentiation of WT- and KO-ESCs towards myogenic fate using the serum-free protocol (Chal et al., 2015). The expression profile shows sequential activation of skeletal muscle progenitor markers (*Pax3* and *Pax7*), MRFs (*Myf5, MyoD, MyoG*) and Differentiation markers (*Myh3, Myh7, Myh8 and Myh2*) in both WT and KO cultures. The gene expression shown is normalized to 18s and heat maps were generated using 2^(−ΔCt)^ values. The data shown in this figure is the mean of 3 replicates.

For spontaneous differentiation, embryoid bodies from iPSCs (WT and KO) were generated and plated on Matrigel-coated dishes differentiated for 21 days. Gene expression profile of skeletal muscle progenitor markers (Pax3 and Pax7), MRFs (Myf5, MyoD, and MyoG) and the expression of various isoforms of myosin heavy chain (Myh3, Myh7, and Myh8) were evaluated on Day 0 (EBs), 7, 14, and 21. As anticipated, the KO-iPSCs showed an enhanced differentiation towards myogenic fate compared to WT-iPSCs (Figure 5A) confirmed by mRNA expression levels of premyogenic and myogenic markers. Following this, by using Chal’s protocol (Chal et al., 2015), we differentiated ESCs (WT and KO) towards the myogenic fate. Both WT- and KO- ESCs were successfully committed towards the myogenic fate, the immunostaining performed on different timepoints confirmed positive expression for markers such as Pax7, Desmin, Myogenin and MF20 (Figure 5B). The quantification of mRNA levels of premyogenic and myogenic markers during directed differentiation of ESCs also revealed an enhanced differentiation in the case of KO cells compared to WT (Figure 5C). In addition to this, the gene expression profile also shows premature activation of potential markers involved in the myogenic program in the case of KO cells compared to WT. Altogether, this data once again proves that the loss of Atoh8 not only affects adult skeletal myogenesis but also embryonic myogenesis.

### 3.6 Atonal homolog 8 regulates myostatin-dependent myoblast proliferation

**FIGURE 6:**
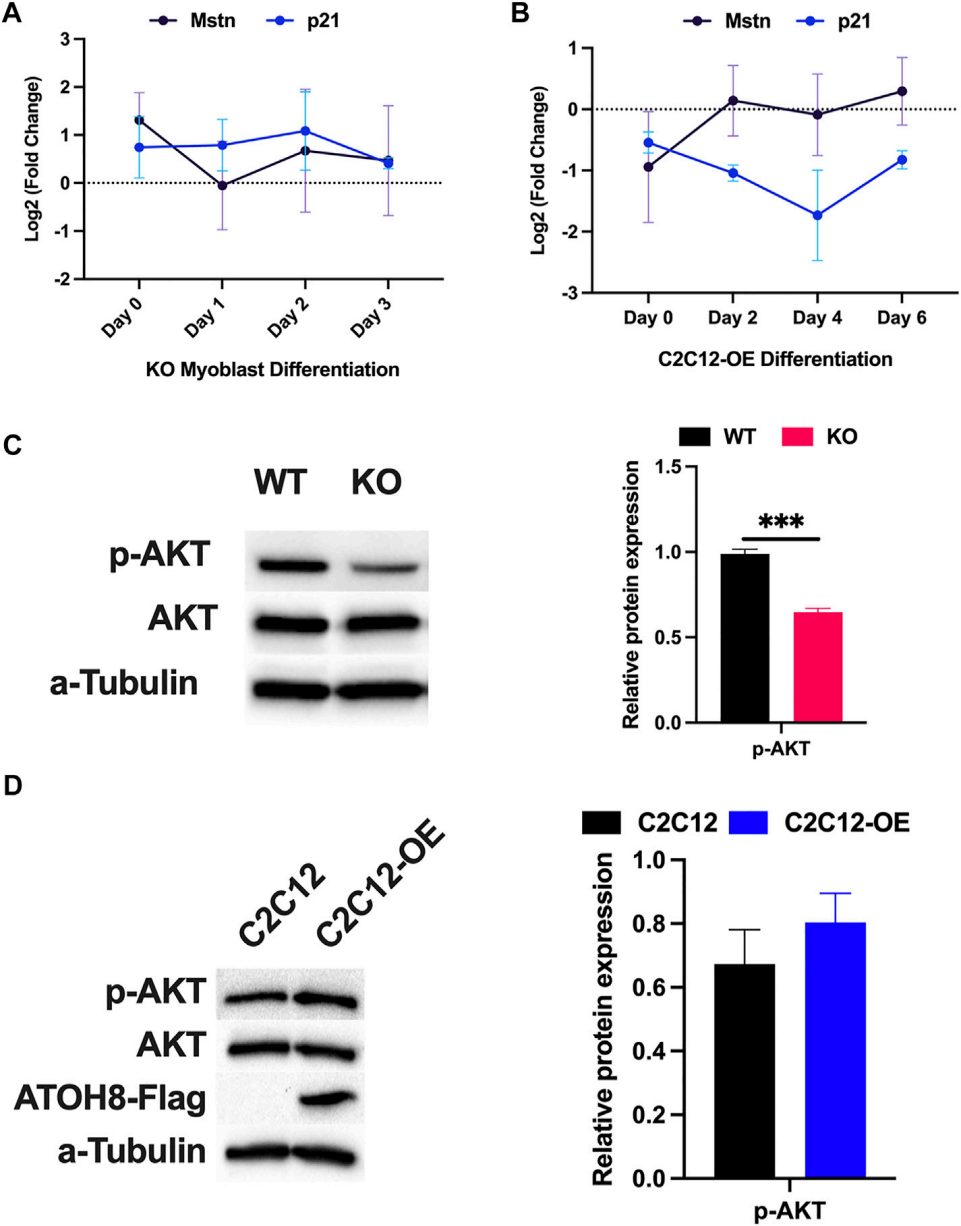
Atoh8 regulates Myostatin-induced AKT/mTOR signaling to promote myoblast proliferation. **(A)** Expression of Myostatin (Mstn) and its downstream target p21 in KO myoblasts during proliferative phase and differentiation. The gene expression data shown are normalized to 18s and relative to WT. **(B)** Expression of Myostatin (Mstn) and p21 in C2C12-OE myoblasts in the proliferative phase as well as during differentiation. The gene expression data shown are normalized to 18s and relative to C2C12 (Control). **(C)** Expression of phosphorylated AKT and AKT proteins is shown on an immunoblot (Left). The quantification of p-AKT showed significantly lower expression in KO primary myoblasts cultured in high serum media compared to WT primary myoblasts, represented in a graph (Right). No change in the expression of AKT was detected. Alpha-Tubulin (a-tubulin) was used as a control for protein quantification. **(D)** Expression of phosphorylated AKT and AKT proteins were shown on an immunoblot (Left). The quantification of p-AKT showed a non-significant increment in C2C12-OE myoblasts cultured in high serum media compared to C2C12 myoblasts, represented in a graph (Right). Expression of Atoh8-Flag protein is shown in the C2C12-OE myoblasts. No change in the expression of AKT has been detected in C2C12 and C2C12-OE myoblasts. Alpha-Tubulin (a-tubulin) was used as a control for protein quantification. The statistical significance was calculated using the Holm-Sidak method using Graphpad. Statistical significance is shown as follows no significance *p* > 0.05, *p ≤ 0.05, **p ≤ 0.01 and ***p ≤ 0.001. The data shown in this figure is the mean ± SEM of 3 replicates.

Myostatin (Mstn) is a member of the TGF-β superfamily and is known for its negative regulatory role in myogenesis. Myostatin was shown to induce a quiescent state in myoblasts by accumulating cells in the G1 and G2 phases of the cell cycle via upregulation of p21 expression (Ríos et al., 2002; Joulia et al., 2003). We have previously found that Atoh8 counteracts TGF-β signaling during reprogramming and in early *in vitro* differentiation of pluripotent stem cells (Divvela et al., 2019). Given the influence of Atoh8 on TGF-β signaling, we evaluated the expression of Myostatin (*Mstn*) and its downstream target *p21* during proliferation and differentiation of primary myoblasts (WT and KO) as well as in C2C12 myoblasts. In the proliferative phase (Day 0), *Mstn* and *p21* were found to be upregulated in KO primary myoblasts compared to control myoblasts (WT). Further during their subsequent differentiation, *Mstn* and *p21* were still observed to be upregulated in KO primary myoblasts (Figure 6A). At the same time in C2C12 myoblasts, following the overexpression of *Atoh8*, the expression of *Mstn* and *p21* were observed to be downregulated in the proliferative phase suggesting that the changes in the rate of myoblast proliferation (WT vs KO and C2C12 vs C2C12-OE) is indeed because of the alterations in the expression of Atoh8. Next, we evaluated the expression of *Mstn* and *p21* during the differentiation of C2C12-OE myoblasts. During differentiation, *Mstn* expression was not observed to change, however, *p21* expression was observed to be downregulated in C2C12-OE myoblasts compared to control (C2C12) (Figure 6B). Together, this data suggests that Atoh8 positively regulates myoblast proliferation via Myostatin signaling either directly or indirectly via modulating its release from cells.

Myostatin was also shown to be capable of inhibiting AKT/mTOR signaling through the Smad2/3 dependent pathway thereby inhibiting protein synthesis to promote muscular atrophy. To check whether the AKT/mTOR signaling pathway is altered in KO myoblasts, we evaluated the expression of AKT and p-AKT at the protein level in primary myoblasts as well as in C2C12 cell lines. To our surprise, we observed a significant downregulation in the phosphorylation of the AKT in KO myoblasts compared to control (WT) with no difference in the expression of AKT (Figure 6C). Confirming this, the overexpression of *Atoh8* has non-significantly upregulated the expression of p-AKT in C2C12-OE cells compared to control (C2C12) with no changes to the expression of AKT (Figure 6D). Altogether, this data suggests that Atoh8 regulates the proliferation and growth of myoblasts.

### 3.7 Atonal homolog 8 knockout mice display poor motor coordination and endurance exercise deficits

**FIGURE 7:**
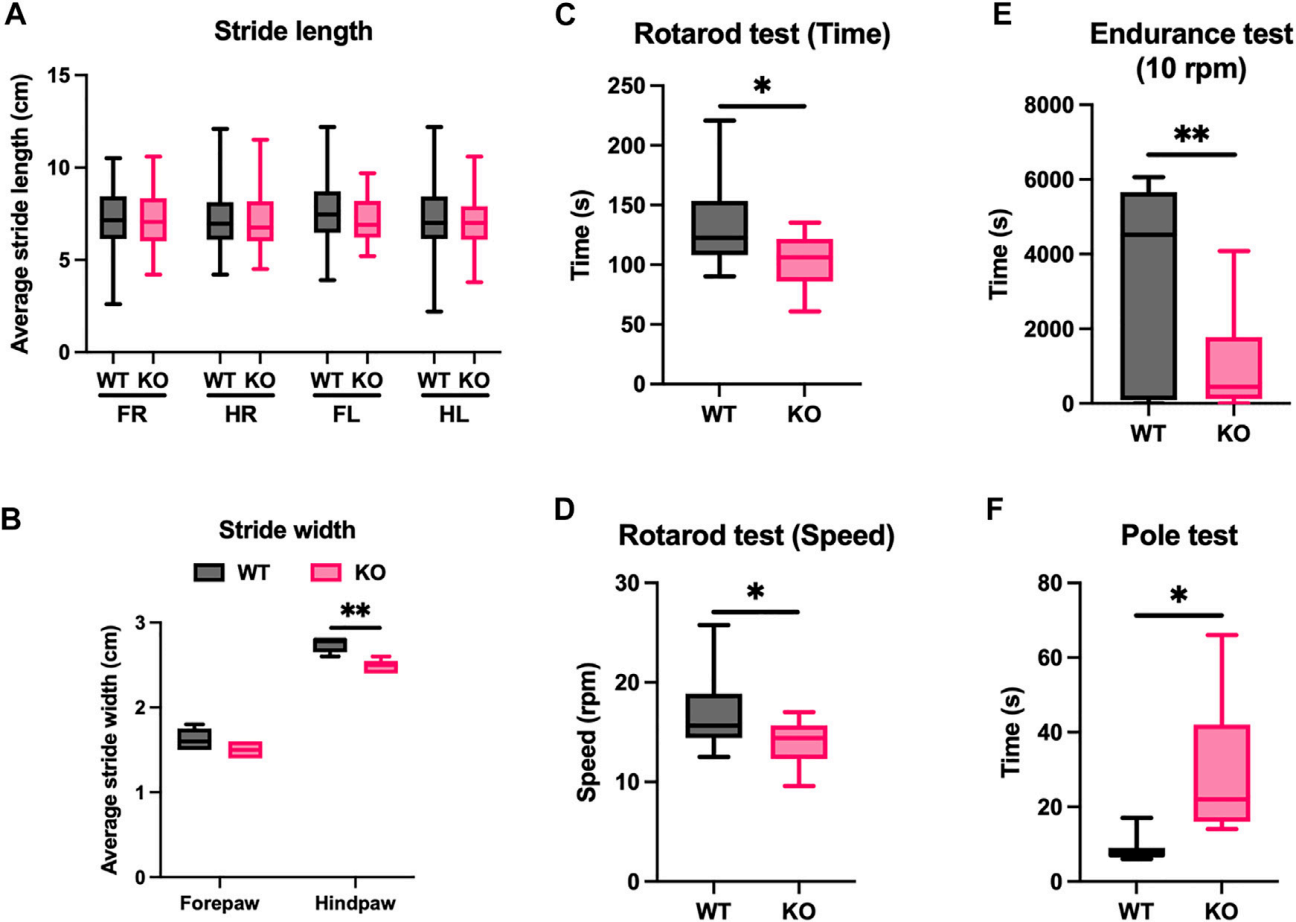
KO mice demonstrate motor coordination and endurance deficits. Comparison of stride length **(A)** and width **(B)** of forepaws and hindpaws in WT and KO mice (FR = Forepaw Right, HR = Hindpaw Right, FL = Forepaw Left and HL = Hindpaw Left). The stride width of hindpaws in KO mice is significantly lower compared to WT suggesting an asymmetric gait. **(C–E)** Rotarod tests show poor motor coordination in KO mice. Time **(C)** and speed **(D)** KO and WT remained on the rotarod. Duration of WT and KO mice endured on the rotarod at a constant speed of 10 rpm **(E)**. **(F)** In the pole test, the KO mice took three times longer than WT to climb down the pole. The statistical significance was calculated using the Holm-Sidak method using Graphpad. Statistical significance is shown as no significance *p* > 0.05, *p ≤ 0.05, **p ≤ 0.01 and ***p ≤ 0.001. The data shown is the mean ± SEM of 3 replicates.

Atoh8 has been described as a pro-neural factor in mice and chicken during retina development. A comprehensive study on the effect of Atoh8 loss on neurogenesis has not been performed. Previous reports indicate that Atoh8 is important for both neurogenesis and skeletal myogenesis (Inoue et al., 2001; Kubo and Nakagawa, 2010; Balakrishnan-Renuka et al., 2013). The transition of fast-glycolytic fibers to slow-oxidative fibers has also been reported to severely affect muscle performance. To investigate whether our Atoh8 knockout mice demonstrate deficits in motor function, we performed a battery of motor function tests such as the footprint analysis, rotarod test, endurance test, pole test, beam walk test and hang wire test to assess motor strength, coordination and endurance in Atoh8 knockout mice in comparison to WT mice. Nine mice (five males and four females) per group at 3 months of age were tested for motor performance. Footprint analysis from KO mice [forepaw right (FR) = 7.164 ± 0.206; hindpaw right (HR) = 7.209 ± 0.236; forepaw left (FL) = 7.248 ± 0.203 and hindpaw left = 7.038 ± 0.225] showed no differences in the stride length compared to WT mice (FR = 7.257 ± 0.275; HR = 7.233 ± 0.243; FL = 7.555 ± 0.270; and HL = 7.207 ± 0.273; Figure 7A). However, the KO mice (2.48 ± 0.037) displayed shorter hind paw stride width compared to WT mice (2.74 ± 0.04), but no difference was observed in the stride width of the forepaws (Figure 7B). Together the footprint analysis showed that KO mice suffer from asymmetric gait. In a standard rotarod test with increasing speed from 4 to 40 rpm, KO mice remained on the rotarod for shorter durations (KO, 106.28 ± 5.58 s; WT, 121.81 ± 7.21 s; Figure 7C) and at lower speeds (KO, 13.78 ± 0.77 rpm; WT, 17.12 ± 1.3 rpm; Figure 7D), indicating poor motor coordination. In an endurance test on the rotarod, KO mice (1,179 ± 310 s) also revealed deficits in endurance training when placed at a constant speed of 10 rpm on the rotarod compared to WT mice (3,392 ± 323 s; Figure 7E). Another test for motor coordination, the pole test, the KO mice (29.43 ± 7 s) took longer to climb down the pole compared to WT (8.57 ± 1.44 s), suggesting a motor coordination deficit in KO mice (Figure 7F). KO mice exhibited similar grip strength compared to WT mice on the hang wire test (Supplementary Figure S6). Additionally, KO mice showed only a tendency to need more time to cross the beam, idle time and hindpaw slips compared to WT mice (Supplementary Figure S6) on the beam walk test. Together these results indicate that the KO mice demonstrate a deficiency in their motor coordination and physical endurance abilities.

### 3.8 Atonal homolog 8 regulates the expression of HIF1a

**FIGURE 8:**
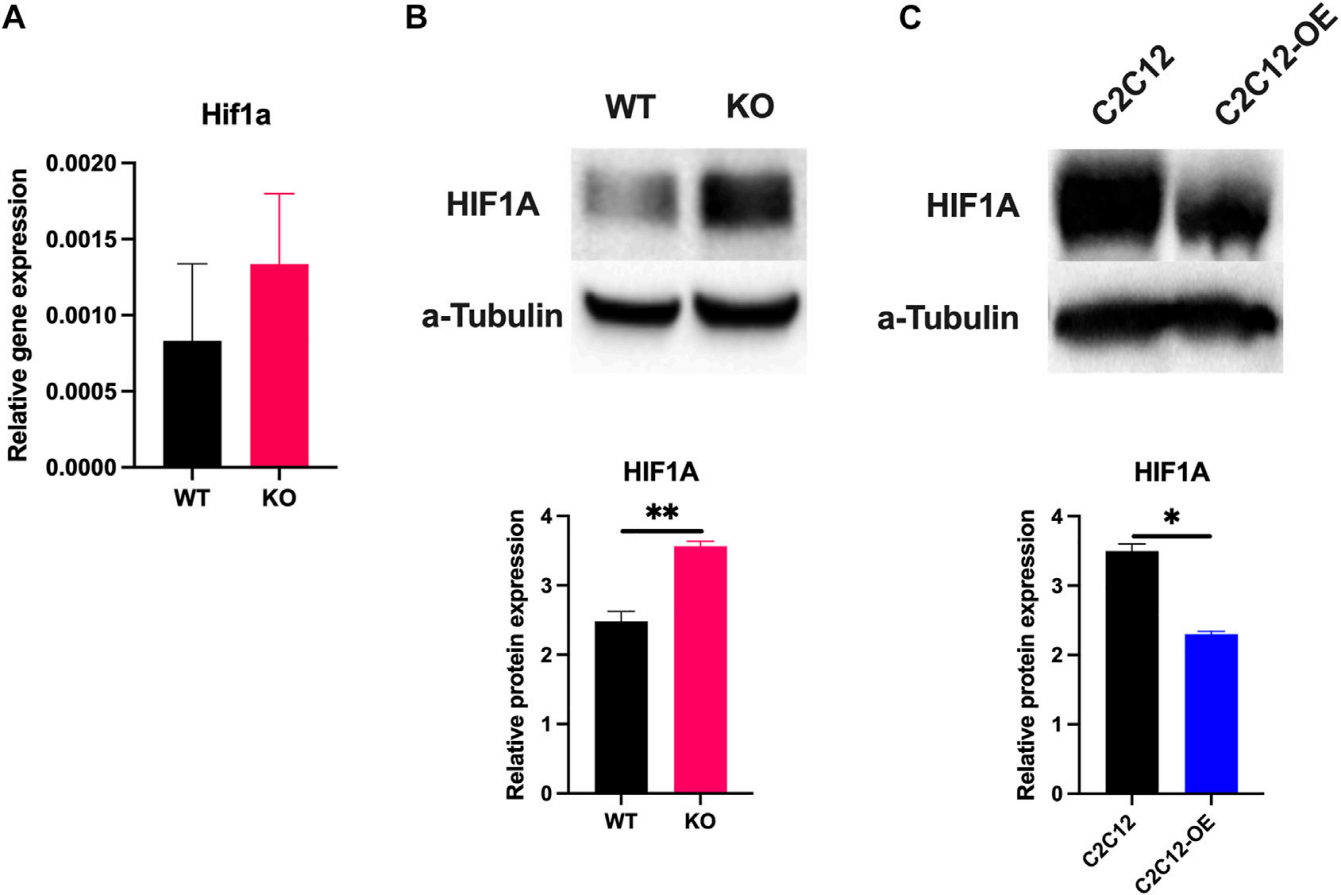
Atoh8 regulates the expression of hypoxia-inducible factor 1a (Hif1a). **(A)** Expression of Hif1a mRNA in WT and KO primary myoblasts. The gene expression shown is normalized to 18s and a bar graph was generated using 2^(−ΔCt)^ values. **(B)** Immunoblot showing significantly higher expression of HIF1A in KO primary myoblasts compared to WT following two hours of incubation with CoCl2. **(C)** Immunoblot showing a significantly lower expression of HIF1A following over-expression of Atoh8 in C2C12-OE myoblasts compared to control (C2C12) following two hours of incubation with CoCl2. The statistical significance was calculated using the Holm-Sidak method using Graphpad. Statistical significance is shown as no significance *p* > 0.05,*p ≤ 0.05, **p ≤ 0.01, and ***p ≤ 0.001. All the experiments shown in this figure are repeated at least three times except for the pole test. The data shown is the mean ± SEM of 3 replicates.

The fast-to-slow fiber type transition of hind limb musculature with an exception of soleus muscle was observed in KO mice (Figures 2B–E) and is also a characteristic feature observed in mice living at higher altitudes, which suffer from hypoxia (Lui et al., 2015; Scott et al., 2015). As observed in KO mice the transition of glycolytic-to-oxidative fibers is also proposed to be an adaptative mechanism developed in rodents to develop endurance and higher oxidative capacity (Fitzsimons et al., 1990). Based on these observations, we have analyzed the expression of another bHLH transcription factor (HIF1a) which plays a central role in the hypoxic response in myoblasts, unlike HIF2a which is predominantly expressed in quiescent satellite cells (Xie et al., 2018). In correlation to a hypoxia-induced muscle fiber phenotype, the KO myoblasts showed a non-significant upregulation of the mRNA levels of *HIF1a* compared to WT myoblasts (Figure 8A). Further to evaluate the expression of HIF1A at the protein level, we quantified the expression of HIF1A following the treatment of myoblasts with CoCl_2_ because of the short half-life of HIF1A. The Western blot analysis of protein lysates from primary myoblasts showed upregulation of HIF1A protein in KO myoblasts compared to WT (Figure 8B). To confirm the above findings, HIF1A protein expression was quantified by Western blot between C2C12 and C2C12-OE cell lines following 2 h of CoCl_2_ treatment. As anticipated, the overexpression of Atoh8 was observed to downregulate the expression of HIF1A suggesting it is a direct or indirect target of Atoh8 (Figure 8C). However, whether the mechanism behind the inactivation of HIF1a by Atoh8 is a genetic or protein-protein interaction is yet to be determined. Altogether, this data suggests that KO mice suffer from hypoxia and as an adaptive mechanism to develop endurance and improve oxidative capacity, an adaptive switch of muscle phenotype from glycolytic fiber type to oxidative fiber type is observed in Atoh8 mutants.

## 4 Discussion

Atoh8 belongs to a large superfamily of transcription factors called bHLH factors. Although it was identified as a pro-neural factor (Inoue et al., 2001), it has been shown to be involved in the development of multiple organs during embryonic development (Ross et al., 2006; Lynn et al., 2008; Böing et al., 2018). Recently, it has also been identified to be a factor that exerts its influence on the transcription factors that maintain a pluripotent state (Song et al., 2015; Divvela et al., 2019). Lately, several studies also reported that changes in the expression of Atoh8 are associated with cancerogenesis (Wang et al., 2015; Ye et al., 2017). Previous research performed in chicken embryos to identify its role in skeletal myogenesis showed that the loss of Atoh8 in ventrolateral lip of the dermomyotome resulted in the complete blockage of differentiation leaving the myoblasts in a predetermined state unable to form the hypaxial myotome (Balakrishnan-Renuka et al., 2013). Later another study which was performed to check whether Atoh8 is involved in skeletal muscle regeneration in humans has shown its expression in satellite cells and at the same time in proliferating myoblasts of the regenerating myofibers suggesting a positive correlation between Atoh8 expression and regeneration (Güttsches et al., 2015). In the current study, we have investigated the role of Atoh8 in adult myogenesis using a constitutive knockout mouse model described previously (Böing et al., 2018) along with WT (*Atoh8* ^*Flag-tag*^) described by our group (Divvela et al., 2019).

The weight of the KO mice is significantly reduced compared to WT which may result from skeletal muscle loss, as skeletal muscle essentially constitutes up to 40% of the body mass. We were interested in understanding how the loss of Atoh8 affects skeletal myogenesis. In a first step, we have evaluated the expression of Atoh8 in the proliferative phase (high serum) and after inducing differentiation (low serum). The expression of Atoh8 was observed to be high in the proliferative phase. However, following the transfer of myoblasts to low serum conditions, the expression of Atoh8 was observed to downregulate as the cells transit from the proliferative phase to the differentiation phase suggesting a positive correlation between Atoh8 and myoblast proliferation. In line with this, Atoh8 expression was previously reported in the myoblasts of the regenerating myofibers (Güttsches et al., 2015). Atoh8 expression was also described to positively correlate with proliferation in bone and cartilage development (Schroeder et al., 2019). To determine if Atoh8 also affects myoblast proliferation, we subjected WT and KO myoblasts to BrdU assay which showed reduced proliferation in the case of KO myoblasts confirming that Atoh8 indeed controls proliferation. Subsequently, KO primary myoblasts showed premature differentiation in presence of high serum conditions. Gene expression analysis of muscle regulatory factors (MRFs) showed an upregulation in KO myoblasts compared to WT. At the same time, in the proliferative phase, the myogenic fusion markers such as Myomixer and Myomaker were also observed to upregulate in KO myoblasts compared to WT. Similarly, when subjected to differentiation, MRFs along with differentiation markers *Myomixer*, *Myomaker,* and *Myh2* were upregulated from day 2 of differentiation. To further confirm this data, we modified the commercially available C2C12 myoblast cell line to stably overexpress the *Atoh8-Flag* sequence with the help of a retrovirus. Following successful generation of the Atoh8 overexpressing C2C12 cell line (C2C12-OE), these myoblasts along with control cells were subjected to BrdU assay, in which we have observed higher proliferation rates compared to control confirming that Atoh8 positively regulates myoblast proliferation. In addition, we analyzed gene expression of MRFs and differentiation markers following overexpression of *Atoh8* in the proliferative phase and differentiation phase. In the proliferative phase, the expression of *Myf5*, *MyoD,* and *Mrf4* remained unaffected. However, the expression of *MyoG* and its downstream fusogenic markers *Mymx* and *Mymk* along with myosin heavy chain (*Myh2*) were found to be downregulated. During differentiation, MRFs except for *MyoG* which was consistently downregulated all through the differentiation (from day 2 to day 6) remained unchanged. Following a similar trend, *Mymx*, *Mymk,* and *Myh2* were also observed to be downregulated in C2C12-OE myoblasts compared to control. Comparing the data from primary myoblasts and C2C12 cells, a strong negative correlation has been observed between the expression of *MyoG* and *Atoh8*. The loss of *Atoh8* resulted in upregulation of *MyoG* whereas, the overexpression of *Atoh8* resulted in downregulation of *MyoG*. Since Atoh8 is a bHLH transcription factor that could bind to the E-Box sequence and MyoG possesses multiple E-box sequences in its promoter, it would be further interesting to check if MyoG is a direct target of Atoh8 (Mastroyiannopoulos et al., 2013).

Confirming the *in vitro* experimental data, the evaluation of cardiotoxin-induced skeletal muscle injury and subsequent regeneration also showed premature onset of differentiation in the case of KO compared to WT. Although the myofibers in the KO mice are thick during early time points of regeneration, the analysis performed at a later time point revealed thin myofibers in KO compared to WT indicating that the KO myoblasts undergo differentiation without generating an adequate number of myoblasts signifying the role of Atoh8 in myoblast proliferation.

Atoh8 has been reported to be responsive to TGF-β and BMP signaling (Kautz et al., 2008; Divvela et al., 2019). In our previous study, in the context of early embryonic differentiation *in vitro*, we have observed that Atoh8 could counteract TGF-β signaling. By keeping this in mind, we have evaluated the expression of *Mstn* and its downstream target *p21* in primary myoblasts. As expected, both *Mstn* and *p21* were found to be upregulated in KO primary myoblasts corroborating their lower proliferation rate in comparison to WT primary myoblasts. At the same time, overexpression of Atoh8 in C2C12 cells showed downregulation of *Mstn* as well as *p21* in the proliferative phase. Fundamentally, the expression of the CDK inhibitor p21 was observed to be consistently high (proliferation and differentiation) in the absence of Atoh8 and low following the overexpression of Atoh8. As *Mstn* expression did not change in compliance with *p21* during differentiation, it can be stated that changes in *Mstn* expression due to the alterations of Atoh8 is restricted to the proliferative phase before the onset of differentiation. Myostatin is known to negatively regulate myogenesis by downregulating *MyoD* and *MyoG* via Smad proteins (Trendelenburg et al., 2009). However, it is not the case in KO primary myoblasts, as *MyoD*, *MyoG* along with *Mstn* are found to be upregulated implying that the activation of *MyoD* and *MyoG* in proliferating KO primary myoblasts is independent of *Mstn* regulation.

In addition to the regulation of myoblast proliferation via p21, Mstn also regulates protein synthesis via the Akt/mTOR pathway by inhibiting the phosphorylation of AKT leading to muscular atrophy (Trendelenburg et al., 2009). In line with these observations, KO myoblasts showed reduced levels of phosphorylation of AKT in KO indicating skeletal muscle atrophy. Although the downstream factors such as FoxO or MuRF1 were not studied in this regard, based on the overall phenotype of the KO skeletal muscle such as lower muscle mass and reduced fiber diameter suggests that the KO mice indeed suffer from muscular atrophy. Confirming the involvement of Atoh8 in the regulation of the Akt/mTOR pathway, overexpression of Atoh8 showed a non-significant increment in the phosphorylation levels of AKT which promotes protein synthesis and muscle growth (Rodriguez et al., 2011). In addition to this, we have also observed that the overexpression of Atoh8 resulted in higher proliferation with delay in the onset of differentiation implying that Atoh8 acts as a limiting factor that helps myoblasts to proliferate in sufficient numbers before the onset of differentiation.

Muscle fiber type analysis also showed an increase in the number of slow-twitch fibers (type -I fibers) in KO mice compared to WT. Such transition of fast-twitch fiber (type IIA, IIX, and IIB) especially from fast-glycolytic phenotype has been observed in KO mice. Previous studies showed a similar transition in the mice that live in higher altitudes in ambient hypoxic conditions (Lui et al., 2015). The transition of fast-twitch fibers to slow-twitch fibers sustained these mice in adapting to hypoxia. Recently, another group studying genes involved in hypoxia has identified Atoh8 as a potential regulator of hypoxia in Tibetan pigs (Wang et al., 2021). At the same time, this study also proposed that Atoh8 has the ability to modulate TGF-β and Akt/mTOR pathways. Morikawa’s group recently showed Atoh8 as a potential regulator of the hypoxic response. Investigations performed in human pulmonary artery endothelial cells (HPAECs) showed that ATOH8 binds to HIF2a and decreases its abundance thereby preventing hypoxic response (Morikawa et al., 2019). Based on the phenotype of the KO mice such as reduced fiber diameter and fast to slow myofiber transition that are associated with hypoxia (Lui et al., 2015), we further evaluated the expression of HIF1A in myoblasts following treatment with CoCl_2_ which is known to induce hypoxia and stabilize HIF1A *in vitro* (Piret et al., 2002). HIF2A is not tested in this context because of its predominant role in satellite cells rather than in myoblasts like HIF1A (Xie et al., 2018). As expected, following 2 h of incubation with CoCl_2_, the expression of HIF1A is observed to be higher in myoblasts derived from KO mice compared to WT. In line with this, the Atoh8 overexpression in C2C12 cells showed reduced levels of HIF1A compared to control cells. It would be further interesting to evaluate, if Atoh8 binds to HIF1A in myoblasts similar to HIF2A in HPAECs and decreases its abundance. Taken together, these data suggest that KO mice suffer from ambient hypoxia.

Multiple studies have so far shown the effect of hypoxia on skeletal muscle. Previous studies have shown that hypoxia has a positive effect on the satellite cells, an increased proliferation of satellite cells was observed in hypoxic conditions with an increase in the expression of Pax7 (Elashry et al., 2022). We previously described an increase in the mesodermal population in KO-ESCs (Divvela et al., 2019), possibly the increase in the Pax7+ population might have also contributed to the enhanced myogenesis that we observed in the KO-ESCs compared to WT-ESCs. Concerning, adult myogenesis, hypoxia was shown to increase satellite cells but not myoblast proliferation or differentiation. Hypoxia was previously shown to inhibit myoblast proliferation and differentiation (Carlo et al., 2004). Although the KO myoblasts showed reduced proliferation, premature differentiation was observed. Recently, two independent groups showed that pre-conditioning of myoblasts with hypoxia resulted in enhanced differentiation (Cirillo et al., 2017; Pircher et al., 2022). Taken together, it can be hypothesized that the KO primary myoblasts seem to suffer from hypoxia *in vivo* and early during their maintenance in cell culture. Subsequently, when KO myoblasts were subjected to differentiation in non-proliferating conditions with low serum the effect of hypoxia is reduced and KO myoblasts might behave like hypoxia pre-conditioned cells.

The KO mice showed poor motor coordination and endurance deficits when challenged. Atoh8 was first identified in the context of neurogenesis as a pro-neural factor (Inoue et al., 2001). It was shown to play an important role in neuronal fate determination, differentiation and maturation in mice and chicken (Inoue et al., 2001; Kubo and Nakagawa, 2010). Its overexpression has been reported to promote neurogenesis. In addition to this, the transition of fast-glycolytic fibers to slow-oxidative fibers that was observed in KO mice has also been reported in spinal and bulbar muscular atrophy (SBMA) which affects both neurons and skeletal muscle by altering their metabolism (Rocchi et al., 2016). Overall, the phenotypes presented in this study concerning KO mice suggest that KO mice suffer from hypoxia which in general alters normal cellular metabolism and neuromuscular health.

## Data Availability

The raw data supporting the conclusion of this article will be made available by the authors, without undue reservation.
