# Supplementary material for: Atonal homolog 8/Math6 regulates differentiation and maintenance of skeletal muscle
Source: Front Cell Dev Biol. 2022 Aug 16;10:950414. doi: 10.3389/fcell.2022.950414 (PMC9438786; doi:10.3389/fcell.2022.950414)
Supplement: Supplementary file 1 [file DataSheet1.docx]

**Supplementary Information**


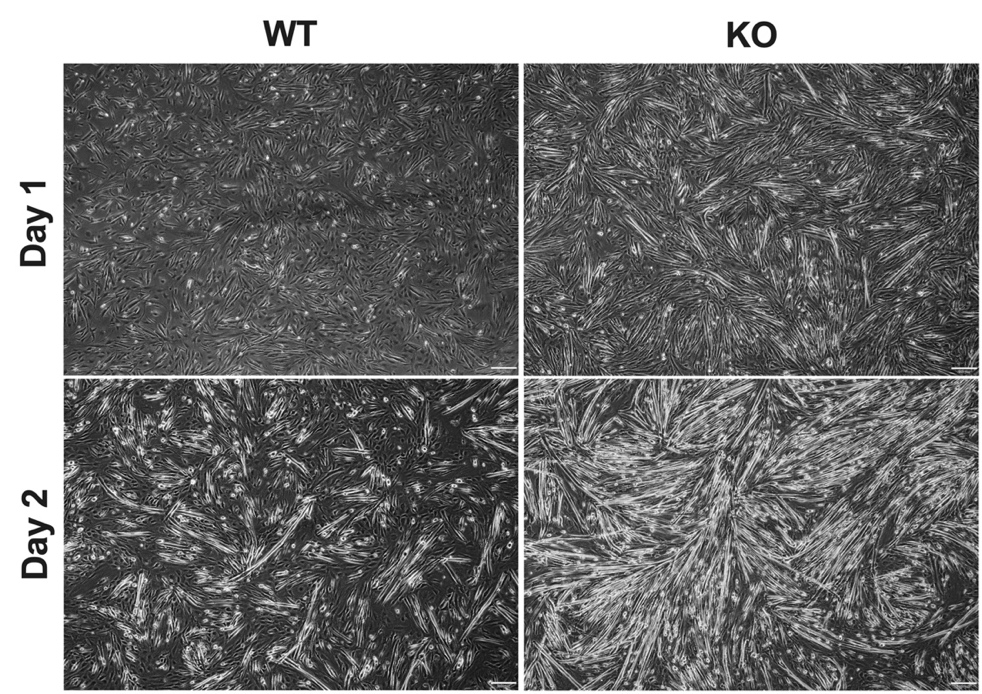


**Figure S1:** Brightfield images of myoblast cultures during myogenic differentiation. KO Primary myoblasts show myotubes already on day 1 after induction of differentiation. Scale bars represent 100 µm.


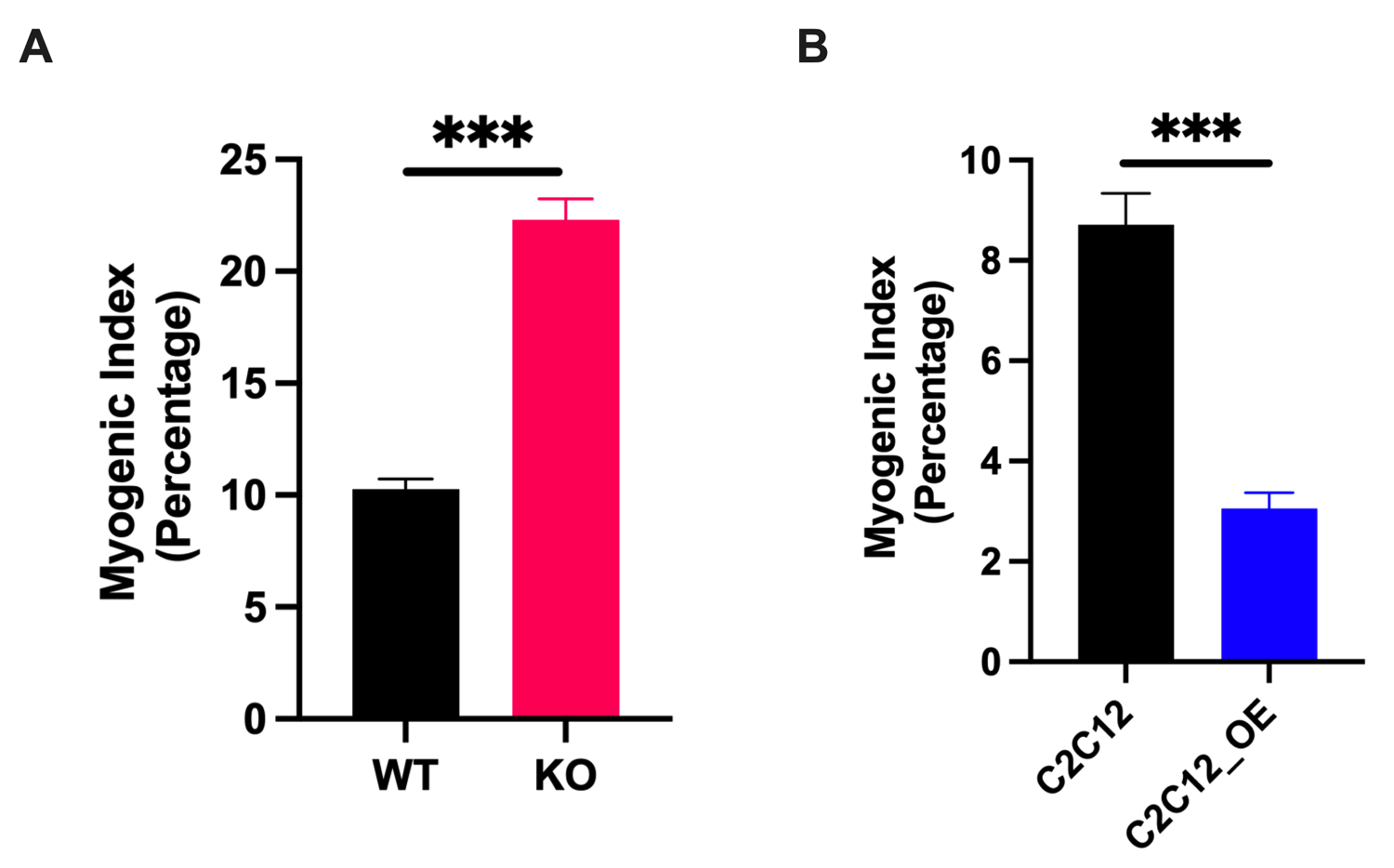


**Figure S2:** Myogenic fusion index in primary myoblast and C2C12 cells. Myogenic fusion index is shown as the percentage of the ratio of a number of nuclei present in a myotube to the total number of nuclei present in the microscopic field following staining with MYH2. **A)** Comparison of fusion index in WT and KO primary myoblasts (p < 0.001) **B)** Comparison of fusion index in C2C12 and C2C12-OE myoblasts (p < 0.001). Changes in the expression of Atoh8 have significantly affected the differentiation and myotube formation. The statistical significance was calculated using the Holm-Sidak method using Graphpad. Statistical significance is shown at (no significance) p > 0.05, * p ≤ 0.05, ** p ≤ 0.01 and *** p ≤ 0.001. All the experiments shown in this figure are repeated at least three times the data shown is the mean±SEM of 3 replicates.


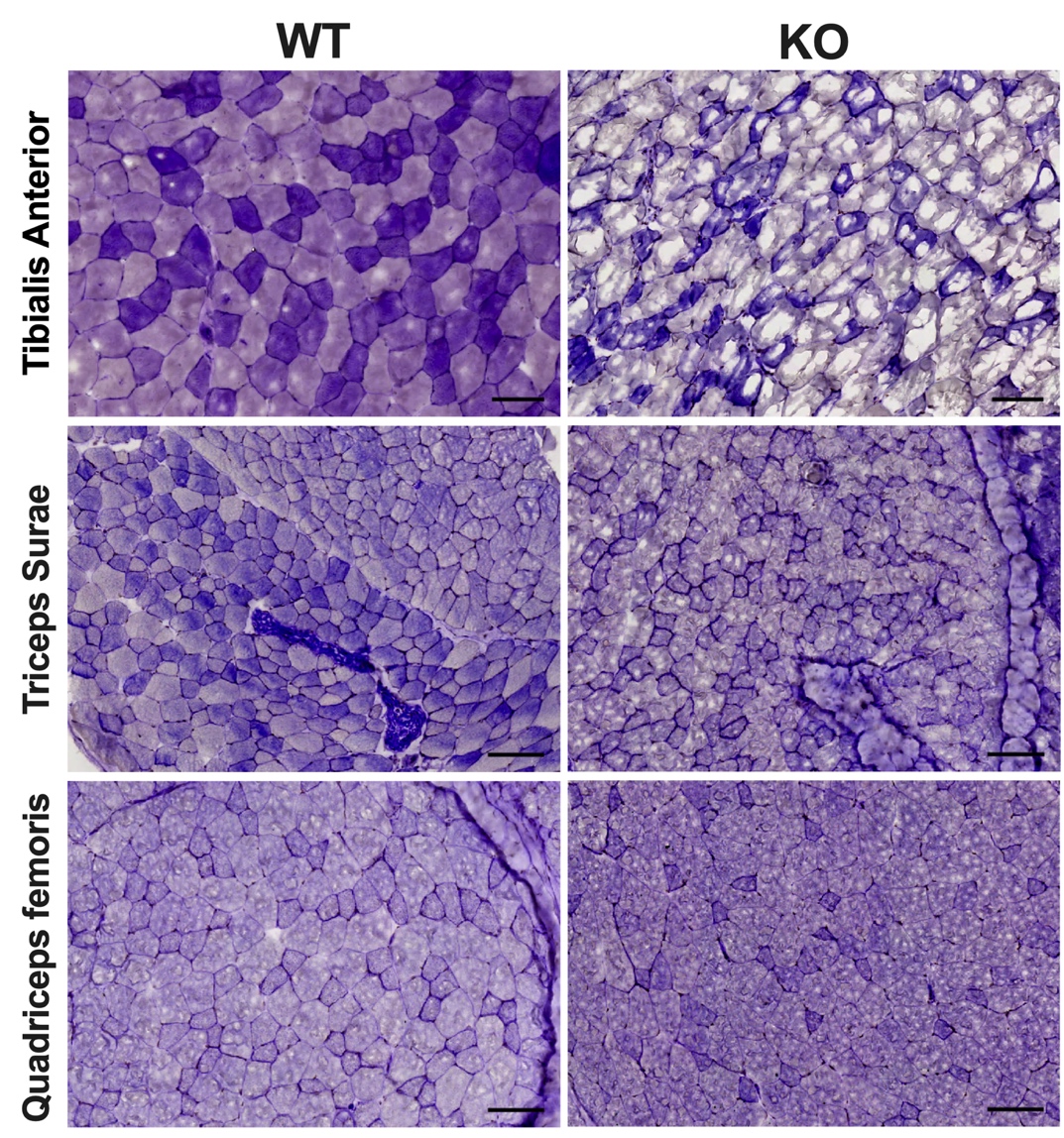


**Figure S3:** PAS staining showing different myofiber types in skeletal muscle sections from WT and KO. Dark-colored muscle fibers indicate higher levels of glycogen compared to weakly stained fibers. The scale bar indicates 100 µm.


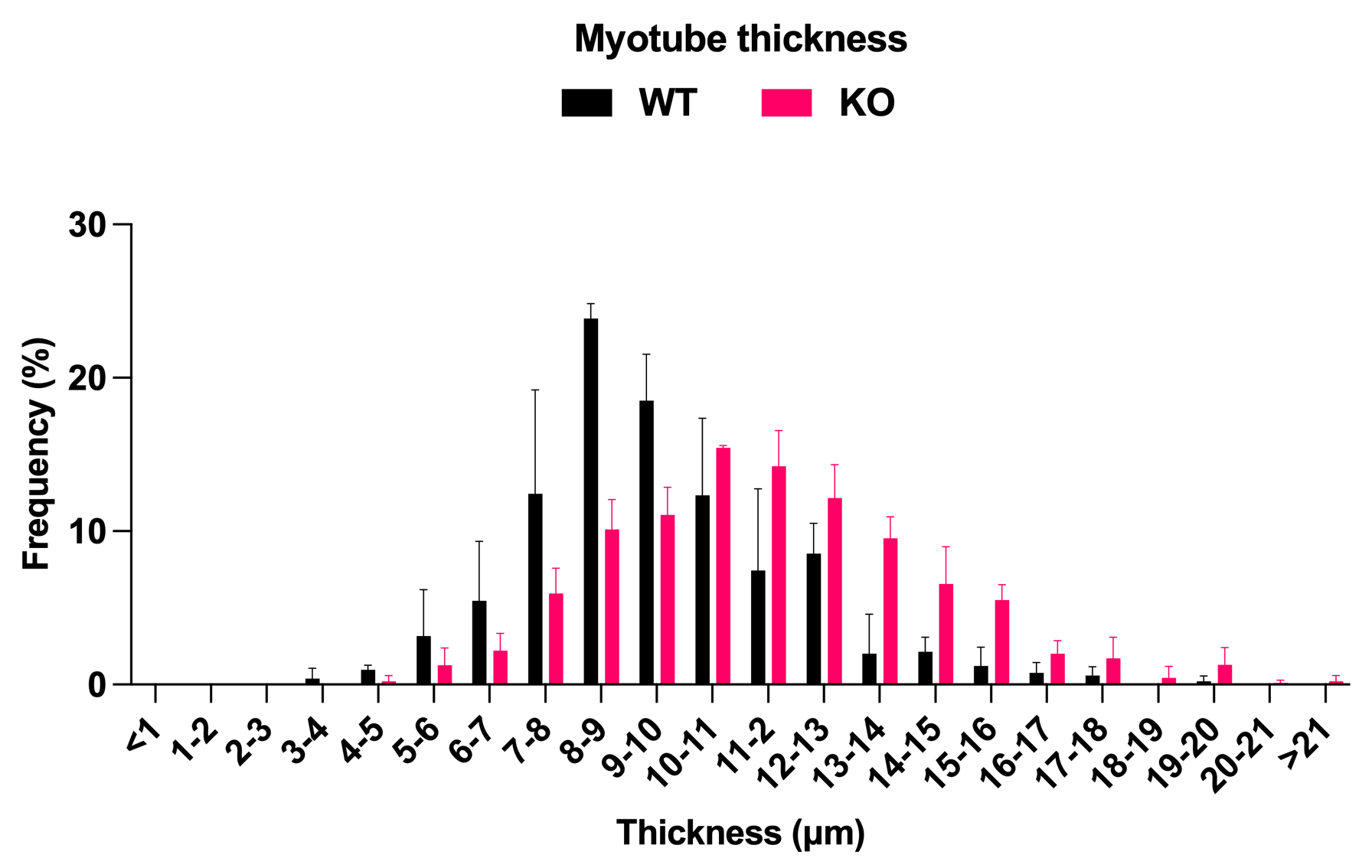


**Figure S4:** Comparison of measurement of the thickness (diameter) of myotubes observed in WT and KO during in vitro differentiation measured using ImageJ on day 3 of differentiation. The data presented here are the mean±SEM of three replicates. The diameter of KO myotubes were observed to be higher compared to WT.


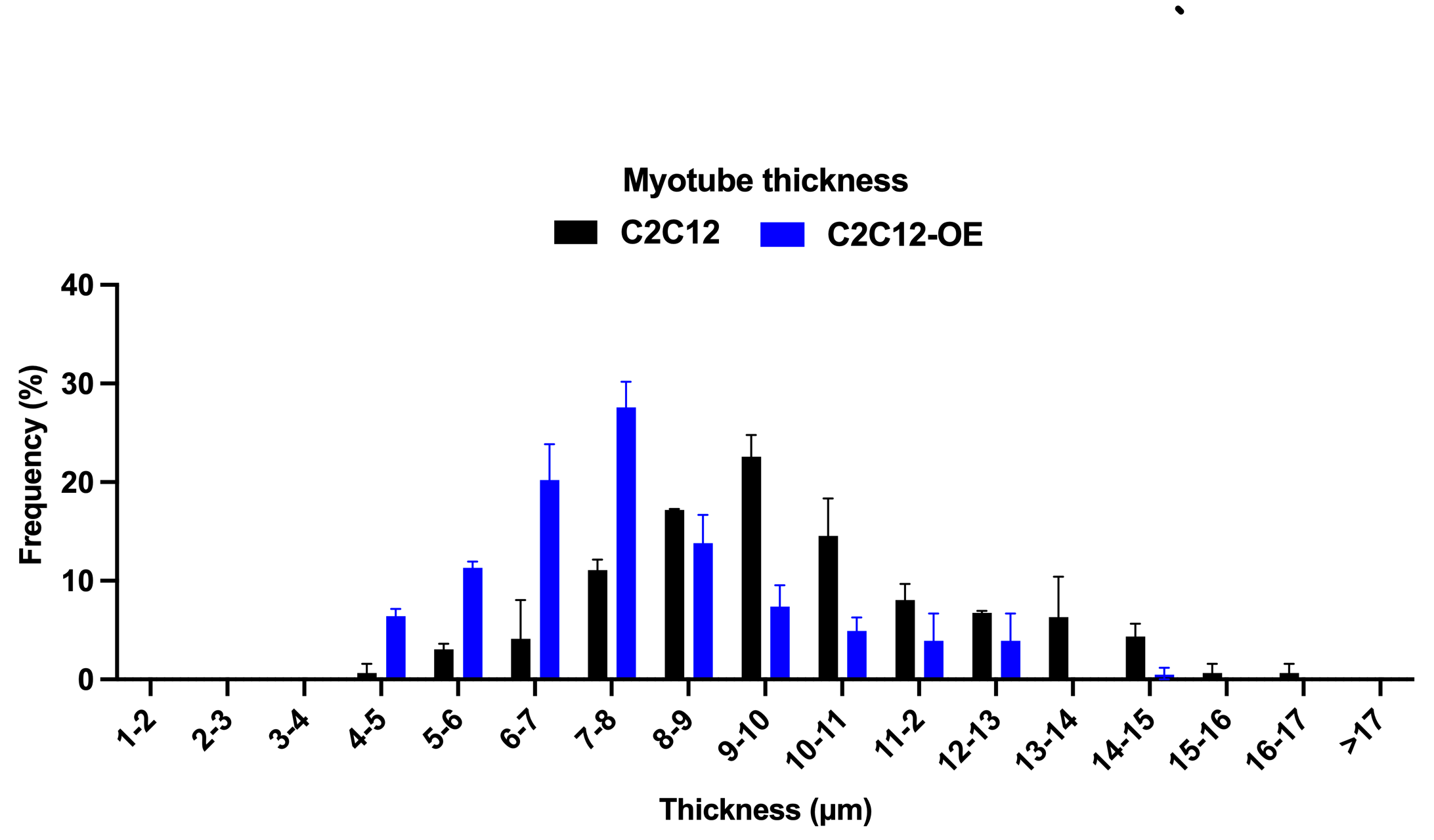


**Figure S5:** Comparison of measurement of the thickness (diameter) of myotubes observed in C2C12 and C2C12-OE cells during in vitro differentiation measured using ImageJ on day 6 of differentiation. The data presented here are the mean±SEM of two replicates. The diameter of C2C12-OE myotubes were observed to be lower compared to C2C12.


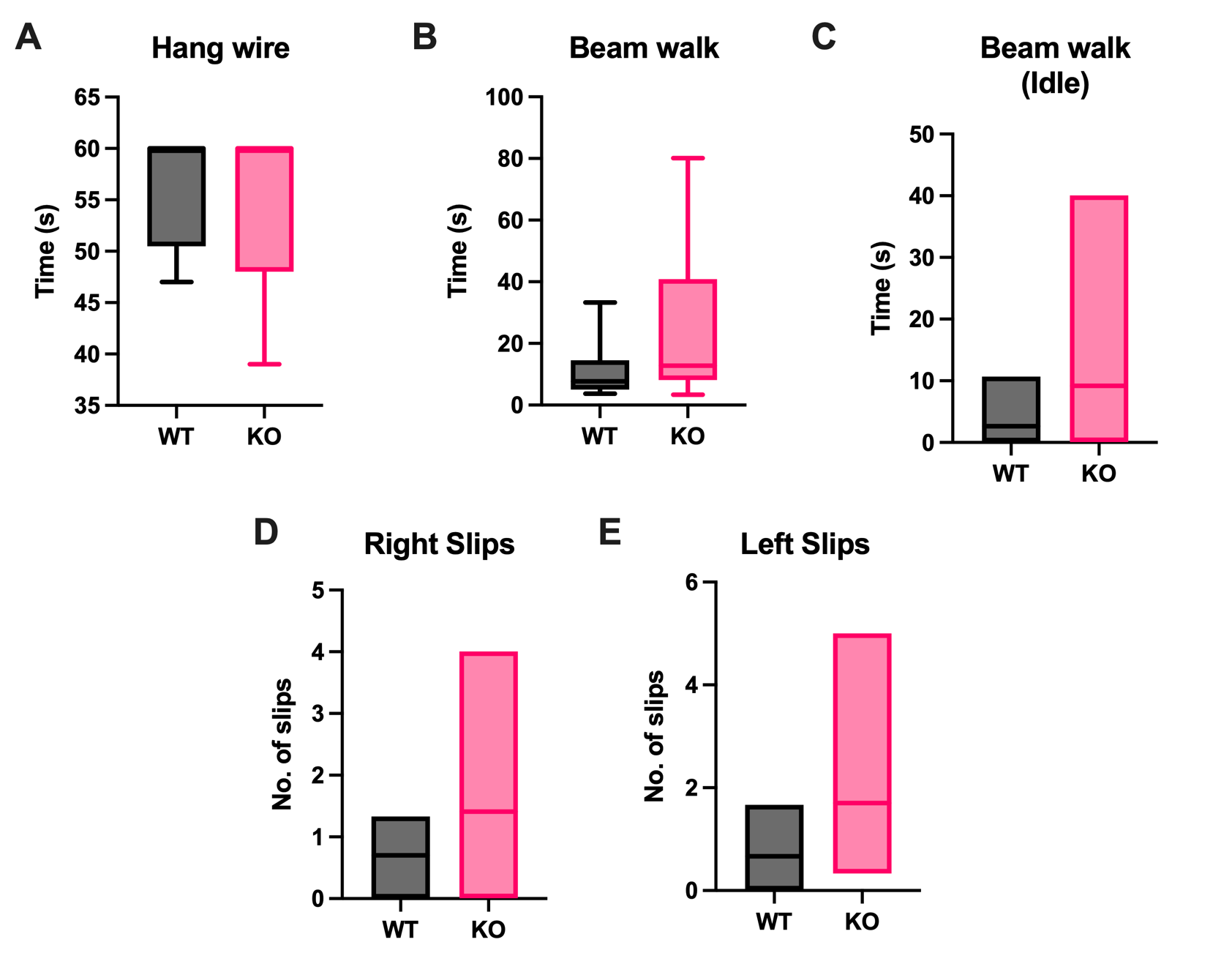


**Figure S6: KO mice behave similarly in the hang wire and beam walk tests for motor coordination compared to WT mice. A)** Comparable performance of WT and KO mice in hang wire test. **B-E)** KO mice showed poor performance in beam walk experiments. The KO mice took a longer time to cross the beam **(B)** and also spent longer idling on beam **(C)** along with more leg slips **(D & E)** compared to WT mice suggesting difficulties in motor coordination. No significance was measured. The statistical significance was calculated using the Holm-Sidak method using Graphpad. All the experiments shown in this figure are repeated at least three times the data shown is the mean±SEM of 3 replicates.


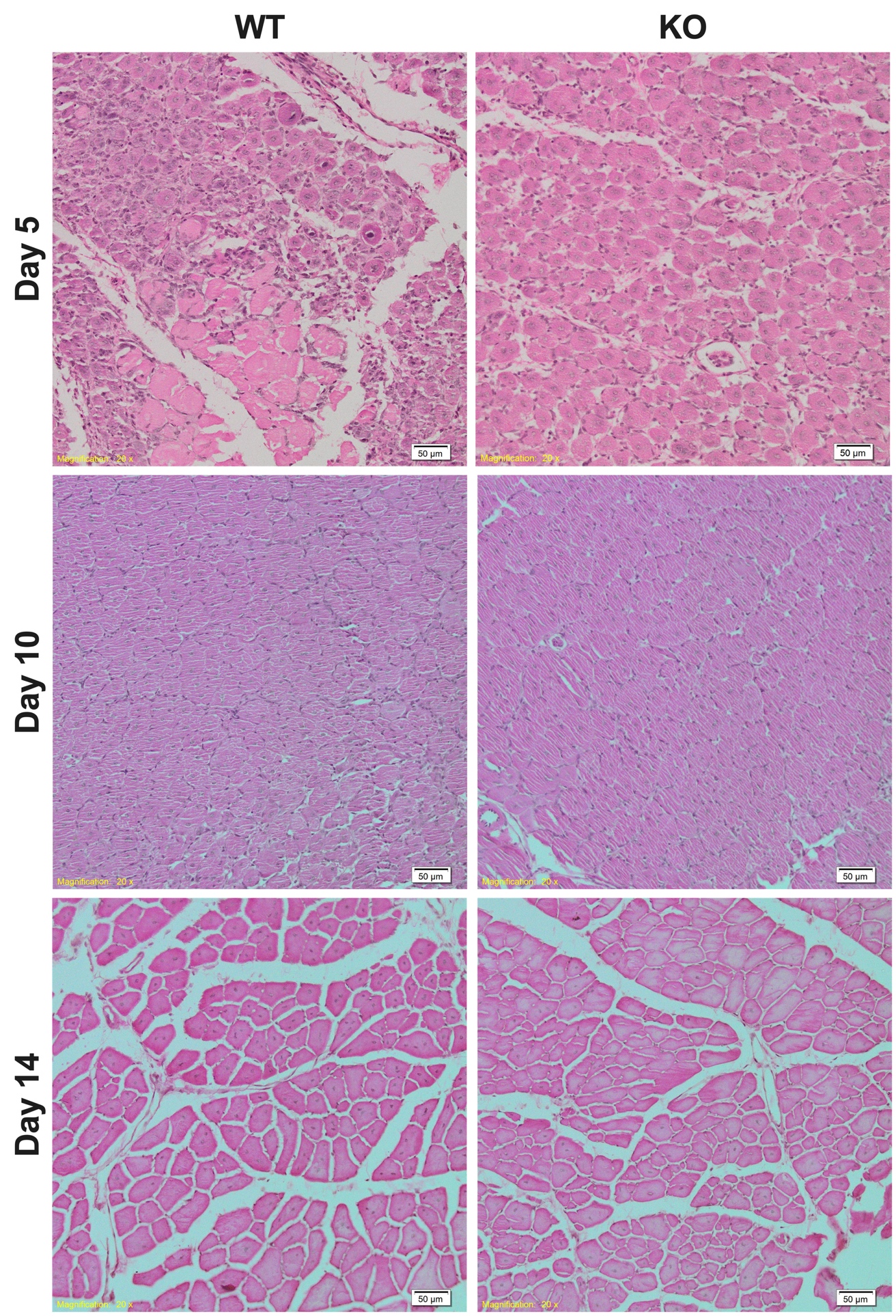


Figure S7: Cardiotoxin induced skeletal muscle regeneration in WT and KO mice. Representative pictures of Hematoxylin & Eosin stained sections of cardiotoxin treated muscles at indicated days following injury. Scale bar represents 50 μM.

**Primer sequences:**

| **Gene** | **Primer Sequence** | **Amplicon size** |
| --- | --- | --- |
| Mstn | Forward: 5' AGTGGATCTAAATGAGGGCAG 3'  Reverse: 5' GTTTCCAGGCGCAGCTTAC 3' | 144 bp |
| Myh3 | Forward: 5' CTTCACCTCTAGCCGGATGGT 3'  Reverse: 5' AATTGTCAGGAGCCACGAAAAT 3' | 107 bp |
| Myh7 | Forward: 5' AATTGTCAGGAGCCACGAAAAT 3'  Reverse:5' GGAGCGCAAGTTTGTCATAAGT 3' | 153 bp |
| Myh2 | Forward: 5' TGCTGCTGATCACCACGAAC 3'  Reverse: 5' TGCTGCTGATCACCACGAAC 3' | 106 bp |
| Myh8 | Forward: 5' CAGGAGCAGGAATGATGCTCTGAG 3'  Reverse: 5' AGTTCCTCAAACTTTCAGCAGCCAA 3' | 113 bp |
| 18s | Forward: 5' GCAATTATTCCCCATGAACG 3'  Reverse: 5' GGCCTCACTAAACCATCCAA 3' | 123 bp |
| Atoh8 | Forward: 5’ GGCAGAAGCTCTCCAAACTG 3’  Reverse: 5’ TCACTCCTTCCGTTTCTTGG 3’ | 179 bp |
| Mrf4 | Forward: 5’ AGAGGGCTCTCCTTTGTATCC 3’  Reverse: 5’ CTGCTTTCCGACGATCTGTGG 3’ | 209 bp |
| Myf5 | Forward: 5’ TGACGGCATGCCTGAATGTA 3’  Reverse: 5’ GCTCGGATGGCTCTGTAGAC 3’ | 173 bp |
| MyoD | Forward: 5’ ACCCGAAGACTGCTGTGTC 3’  Reverse: 5’ GTCTGACGATTCACAACAGGC 3’ | 208 bp |
| MyoG | Forward: 5’ GCAATGCACTGGAGTTCG 3’  Reverse: 5’ ACGATGGACGTAAGGGAGTG 3’ | 98 bp |
| Mymx | Forward: 5’ TTCCTCCCGACAGTGAGCAT 3’  Reverse: 5’ TGCGATCTGACTGGTGTCTCC 3’ | 175 bp |
| Mymk | Forward: 5’ AGACTTCCGTGACTCCTACCAG 3’  Reverse: 5’ GCACAGCACAGACAAACCAG 3’ | 98 bp |
| P21 | Forward: 5' TCACTCTGTGTGTCTTAATTA 3'  Reverse: 5' AGGACTGTTCCTCCGGTATAGG 3' | 259 bp |

**List of antibodies**

| **Antibody** | **Company** | **Application** | **Concentration** |
| --- | --- | --- | --- |
| BrdU | Abcam (ab6326) | ICC | 1:500 |
| Desmin | Dako (M0760) | ICC | 1:200 |
| MYH2 | DSHB (SC-71) | ICC | 5 μg/ml |
| MYH4 | DSHB (BF-F3) | ICC | 5 μg/ml |
| MYH7 | DSHB (A4.840) | ICC | 5 μg/ml |
| Myogenin | DSHB (F5D) | ICC | 5 μg/ml |
| Laminin | Sigma Aldrich (L9393) | ICC | 1:200 |
| Pxa7 | DSHB | ICC | 5 μg/ml |
| MF20 | DSHB | ICC | 5 μg/ml |
| AKT | Cell Signaling Technology (2920S) | Western blot | 1:1000 |
| p-AKT | Cell Signaling Technology (9271S) | Western blot | 1:1000 |
| Anti-Flag | Sigma Aldrich (F1804) | Western blot | 1:500 |
| a-Tubulin | Sigma Aldrich (T9026) | Western blot | 1:1000 |
| Goat Anti-rabbit IgG conjugated with HRP | Cell signaling Technology (7074) | Western blot | 1:10000 |
| Rabbit Anti-mouse IgG conjugated with HRP | Jackson Immuno Research (315-035-008) | Western blot | 1:10000 |
| Donkey Anti-rabbit IgG, Alexa 488 | Invitrogen (A21206) | ICC | 1:1000 |
| Goat Anti-rabbit IgG, Alexa 568 | Invitrogen (A11031) | ICC | 1:1000 |
| Goat Anti-mouse IgG, Alexa 488 | Invitrogen (A11001) | ICC | 1:1000 |
| Donkey Anti-mouse IgG, Alexa 568 | Invitrogen (A10037) | ICC | 1:1000 |
| Goat Anti-mouse IgM, Alexa 647 | Jackson Immuno Research (115-605-020) | ICC | 1:100 |
| Goat Anti-mouse IgG, Alexa 421 | Jackson Immuno Research (115-675-205) | ICC | 1:200 |
